# Why *Wolbachia*-induced cytoplasmic incompatibility is so common

**DOI:** 10.1073/pnas.2211637119

**Published:** 2022-11-07

**Authors:** Michael Turelli, Andrew Katznelson, Paul S. Ginsberg

**Affiliations:** ^a^Department of Evolution and Ecology, University of California, Davis, CA 95616

**Keywords:** levels of selection, epidemiology, spite, mutualism, reproductive manipulation

## Abstract

Cytoplasmic incompatibility (CI) is the most common reproductive manipulation produced by *Wolbachia*, obligately intracellular alphaproteobacteria that infect approximately half of all insect species. Once infection frequencies within host populations approach 10%, intense CI can drive *Wolbachia* to near fixation within 10 generations. However, natural selection among *Wolbachia* variants within individual host populations does not favor enhanced CI. Indeed, variants that do not cause CI but increase host fitness or are more reliably maternally transmitted are expected to spread if infected females remain protected from CI. Nevertheless, approximately half of analyzed *Wolbachia* infections cause detectable CI. Why? The frequency and persistence of CI are more plausibly explained by preferential spread to new host species (clade selection) rather than by natural selection among variants within host populations. CI-causing *Wolbachia* lineages preferentially spread into new host species because 1) CI increases equilibrium *Wolbachia* frequencies within host populations, and 2) CI-causing variants can remain at high frequencies within populations even when conditions change so that initially beneficial *Wolbachia* infections become harmful. An epidemiological model describing *Wolbachia* acquisition and loss by host species and the loss of CI-induction within *Wolbachia* lineages yields simple expressions for the incidence of *Wolbachia* infections and the fraction of those infections causing CI. Supporting a determinative role for differential interspecific spread in maintaining CI, many *Wolbachia* infections were recently acquired by their host species, many show evidence for contemporary spatial spread or retreat, and rapid evolution of CI-inducing loci, especially degradation, is common.

## Significance

*Wolbachia* are obligately intracellular alphaproteobacteria that infect approximately half of all insect species. Maternal inheritance of these endosymbionts produces selection to enhance female fitness. In addition to mutualistic phenotypes such as nutrient provisioning, *Wolbachia* produce various reproductive manipulations that favor infected females. Most common is cytoplasmic incompatibility, namely reduced embryo viability when *Wolbachia*-infected males fertilize *Wolbachia*-uninfected females. The regular loss of cytoplasmic incompatibility indicates this phenotype is not favored by natural selection among *Wolbachia* variants within host populations. Instead, we argue that cytoplasmic incompatibility is pervasive because it enhances interspecific transmission and intraspecific persistence. Specifically, cytoplasmic incompatibility produces high prevalence frequencies within host populations and allows *Wolbachia* to persist in host species even when their mutualist phenotypes wane or vanish.

*Wolbachia*, maternally inherited alphaproteobacteria, may be the most common animal endosymbiont, occurring in about half of all insect species as well as other arthropods and nematodes (1). Relatively few *Wolbachia* infections of arthropods have been characterized for reproductive manipulation or any other effects, but among those tested, approximately half cause cytoplasmic incompatibility (CI) (e.g., see ref. 2 for *Drosophila* data). CI is defined by elevated embryo mortality when uninfected ova are fertilized by sperm from *Wolbachia*-infected males (3). CI intensity (i.e., the fraction of embryos killed) varies from a few percent to 100% and depends on *Wolbachia* genotype, host genotype, and various conditions, including temperature and host age (4–7). CI can also occur in matings of males and females carrying incompatible *Wolbachia* variants (8–10). CI was first described in the mosquito *Culex pipiens* and its close relatives (9, 11). The pioneering work of Beckmann and Fallon (12) on a *Wolbachia* protein found in *Culex* sperm initiated progress toward identifying pairs of loci that underlie CI in many taxa (reviewed in refs. 13–15). Our analyses address the evolutionary forces determining the prevalence of CI-causing *Wolbachia*. Although initially associated with *Wolbachia* (9, 16), other maternally inherited microbes also produce CI (17–21). Our analyses apply to all such microbes, but we focus on *Wolbachia* because its population biology, molecular biology, and patterns of acquisition are more completely characterized.

The prevalence of CI-causing *Wolbachia* presents a puzzle. As noted by Prout (22) and Turelli (23), natural selection among mutually compatible *Wolbachia* variants in a host species does not favor CI. As first proposed by Hurst and McVean (24), the prevalence of CI may be more plausibly explained by a process of clade selection in which CI-causing *Wolbachia* lineages are more likely than non-CI-causing lineages to spread to new host species. Consistent with the data then available (e.g., see refs. 24–26), Hurst and McVean (24) assumed that *Wolbachia* infections generally decrease host fitness. This now seems doubtful, with increasing evidence, reviewed below, suggesting that many *Wolbachia* infections are mutualistic. We generalize the Hurst and McVean (24) clade-selection hypothesis, showing that both mutualistic and deleterious *Wolbachia* variants are more likely to spread to new host species if they induce CI. In support of this hypothesis, we review data indicating that many *Wolbachia* infections are relatively young (originating on the order of tens of thousands of years ago, long after speciation), that spatial and temporal *Wolbachia* frequencies within species often vary, and that *Wolbachia* regularly lose the ability to induce CI while retaining the ability to resist it. These observations suggest regular turnover of *Wolbachia* infections within and among host species.

Hurst (27) proposed that natural selection would favor increased CI, but this conjecture was refuted by algebraic analyses of the fate of *Wolbachia* variants within individual host populations (22, 23) and metapopulations (28). Those analyses focused on mutually compatible variants that differ in the intensity of CI produced by matings of infected males to uninfected females (i.e., the average fraction of embryos that die because of incompatibility), the fidelity of *Wolbachia* maternal transmission, and the relative fitness (specifically viability and fecundity) of infected versus uninfected females. Within host populations, there is no selection among *Wolbachia* variants for increased CI. Specifically, among mutually compatible *Wolbachia* variants within a population (i.e., females carrying each variant are immune to the CI-inducing effects of the others), natural selection favors the variant whose female carriers produce the largest number of *Wolbachia*-infected progeny (i.e., product of relative fecundity times fraction of offspring that carry the infection). This is true irrespective of whether males carrying the favored variant produce CI when mated to uninfected females (23). Metapopulation structure, namely small local populations linked by migration, produces weak selection for CI, but very small positive effects on relative fitness (i.e., increases on the order of 10^−3^) generally suffice to overcome the intergroup selection advantage associated with even strong CI (28). Consistent with this prediction, several studies of *Wolbachia* infections in a wide range of hosts indicate relatively recent loss of function for the loci that cause CI [but typically not loss of functional loci that protect hosts from CI (15, 29, 30)].

Because very closely related *Wolbachia* (separated by 1,000 to 10,000 y) infect distantly related, reproductively isolated host lineages (separated by 1 My to 10 My, e.g., refs. 31–33), processes both among and within host lineages can contribute to differential proliferation of *Wolbachia* variants across the tree of life (24). Recent data, reviewed below, indicate relatively rapid movement of *Wolbachia* lineages between host species by a combination of both introgression between closely related species and nonsexual horizontal transmission between more distantly related hosts. Nonsexual horizontal transmission can be mediated by both parasitoids (34) and host plants (35). The turnover of *Wolbachia* within host species often seems to occur much faster than the timescale of the origin and extinction of host species (32). Hence, to understand *Wolbachia* evolution, we must consider the frequency dynamics of variants both within individual host species and among host species, specifically the rate of spread to new host species, the duration of typical *Wolbachia*–host associations, and the persistence of CI within *Wolbachia* lineages. Debates concerning the relative importance of levels of selection often emphasize discordant selection at different levels (e.g., natural selection within groups may favor selfish behavior, but selection among groups may favor groups with more altruists) (36–38). Understanding CI evolution across *Wolbachia* lineages is simplified by the fact that there is essentially no selection for or against CI among *Wolbachia* lineages within individual host species (22, 23, 28). Hence, the maintenance and evolution of CI are plausibly determined by relative movement of *Wolbachia* lineages among host species and the persistence of *Wolbachia* infections and CI induction within host species.

This interspecific versus intraspecific transmission perspective is explicit in the analyses of *Wolbachia* pervasiveness by Hurst and McVean (24) and Werren and Windsor (39). Building on the work of Turelli (23) and Prout (22), Hurst and McVean (24) proposed a “reversible evolution” model for CI in which CI-causing *Wolbachia* invade an uninfected host but are displaced by non-CI-causing variants (resistant to CI), which are then outcompeted by more fit *Wolbachia*-uninfected cytotypes. This cycle assumes that CI-causing variants impose a greater fitness cost on hosts than non-CI-causing variants, which are implicitly assumed to also reduce host fitness. Hurst and McVean (24) argued that the *Wolbachia* variants that persist among insect species are those best able to invade new host species through horizontal transmission. Their analyses suggest that deleterious CI-causing *Wolbachia* persist because CI facilitates invasion of new hosts. We generalize this framework to consider both mutualistic and deleterious *Wolbachia*, motivated by data suggesting that many, and plausibly most, natural *Wolbachia* infections are mutualistic, whether or not they induce CI (2, 3, 30, 40–42).

Initial field and laboratory studies suggested that *Wolbachia* might generally reduce host fitness, specifically fecundity (25, 26). As illustrated by Eq. **1**, direct fitness effects dominate the dynamics of rare *Wolbachia* infections, whether or not they cause CI, because CI is effectively nonexistent when *Wolbachia*-infected males are very rare. The deleterious-*Wolbachia* paradigm is demonstrably correct for *Wolbachia* transinfections (i.e., *Wolbachia* experimentally transferred from one host species to another) that are being used to control insect-vectored diseases of humans (43–45) and plants (46). For these systems, there is an unstable equilibrium frequency that CI-causing variants must exceed before their frequencies tend to increase deterministically through the frequency-dependent advantage associated with CI (Eq. **1**). Once established locally, these infections with bistable dynamics can spread spatially (25, 47). But initial local establishment requires purposeful introduction (48, 49) or a genetic drift–like sampling process that gets local frequencies above the unstable equilibrium (50, 51).

The Hurst and McVean (24) assumption that naturally occurring, CI-causing *Wolbachia* are generally deleterious no longer seems plausible. The paradigm shift is based on several observations concerning temporal and spatial variation of *Wolbachia* frequencies in nature. First, the rate of spatial spread of the CI-causing *w*Ri *Wolbachia* in both California and Australian *D. simulans* populations was on the order of 100 km/y (25, 40). This makes sense only if long-distance, human-mediated dispersal can initiate local spread starting from very low frequencies. Bistability produced by deleterious *Wolbachia* effects precludes this. Indeed, for *Wolbachia* transinfections that are demonstrably deleterious, such as *w*Mel introduced from *D. melanogaster* into *Aedes aegypti*, spatial spread is orders of magnitude slower (on the order of 100 m/y for *Ae. aegypti* rather than 100 km/y for *D. simulans*), despite comparable dispersal distances and generation times for *D. simulans* and *Ae. aegypti* (43). Second, the non-CI-causing *Wolbachia* variant *w*Au was observed to spread through Australian *D. simulans*; this makes sense only if *w*Au is mutualistic (40). Third, many *Wolbachia* that cause little or no CI, or other detectable reproductive manipulation, persist in natural populations [e.g., the variants *w*Mel in *D. melanogaster* (52, 53); *w*Suz in *D. suzukii* (3); *w*Mau in *D. mauritiana* (30); and the *Wolbachia* in the three-species *D. yakuba* clade (54, 55)]. Fourth, we now have several plausible examples of direct fitness benefits associated with *Wolbachia*, including protection from viruses (e.g., refs. 56–58), nutritional provisioning (e.g., refs. 59 and 60) and various life history effects (61). The temporal and spatial frequencies of *Wolbachia* infections that cause little or no CI seem most compatible with a balance between positive fitness effects (many of which remain to be identified) and imperfect maternal transmission (53, 62). Because of maternal transmission, we expect *Wolbachia* to evolve toward mutualism within host lineages (23), and this has been observed over a timescale of decades (63). Hence, it now seems likely that many *Wolbachia* invade new hosts through mutualism rather than reproductive manipulation. Although CI is not favored within individual host species, we argue that CI enhances spread among host species for both mutualistic and deleterious *Wolbachia*.

The pervasiveness of CI-causing *Wolbachia* can be understood by analogy to the spread of disease microbes within and among conspecific individuals. This epidemiological perspective on the *Wolbachia* pandemic among insects was invoked by Werren and Windsor (39) to explain the relative constancy of the fraction of insect species infected (*Wolbachia* “incidence”) across continental regions. Their model considered only a transmission rate to new host species (*T*) and a loss rate for infections in host species (*L*). We extend their model by considering the relative transmission and loss rates for *Wolbachia* variants that do or do not cause CI, allowing for loss of functional CI loci within *Wolbachia* lineages (15, 29, 30). Simple models illustrate that CI both increases the transmission rate, *T*, and decreases the loss rate, *L*.

Epidemiological models, which focus on disease-causing microbe density within host individuals and frequency among conspecific hosts, can be adapted to illuminate the incidence of alternative *Wolbachia* forms among host species. For instance, among disease microbes, if variants provide immunity to one another, competition favors the variant with the largest *R*_0_, corresponding to “the average number of secondary cases arising from an average primary case in an entirely susceptible population” (64, p. 20). This corresponds to selection among mutually compatible *Wolbachia* variants favoring a higher *T* and longer persistence time within each host species. For disease microbes, a classical explanation for the evolution of intermediate virulence, as exemplified by myxoma in Australian rabbits (65), is that there is often a tradeoff between transmission rate and infectious duration (64, 66). For example, increased myxoma titer may increase transmission but accelerate host death. In contrast, no comparable tradeoff, now between *Wolbachia* frequencies within host species and the duration of *Wolbachia* infections within those host species, is expected for *Wolbachia* variants that cause CI. As discussed below, CI-causing *Wolbachia* variants are expected to be at higher frequencies within host species (producing a higher transmission rate between species) and also to persist longer in their host species than non-CI-causing variants. We illustrate both ideas with simple calculations and simulations. Because so much *Wolbachia* biology remains unknown, our goal is not to produce a fully parameterized model that predicts the frequency of alternative *Wolbachia* forms across all insects (or potential arthropod hosts) but simply to present a plausible hypothesis explaining why CI is so prevalent.

## Theoretical Framework

### Deterministic Analyses of *Wolbachia* Frequencies within Host Species.

Our intraspecific analyses build on a simple discrete-generation, deterministic model for *Wolbachia* frequency dynamics (67). The model has been used to explore evolutionary dynamics (23) and to address the consequences of positive *Wolbachia* effects on host fitness (53). It incorporates imperfect maternal transmission, CI, and the effects of *Wolbachia* on host fitness, modeled as differential fecundity (3). We assume that, on average, a fraction μ of the ova produced by an infected female are uninfected, and that uninfected ova from infected females are as susceptible to CI as are ova from uninfected females (see ref. 68 for empirical support in *D. simulans*). Embryos produced from fertilizations of uninfected ova by sperm from infected males hatch with frequency *H* = 1 − *s*_h_ relative to the fraction of embryos that hatch from the three compatible types of fertilizations, all of which are assumed to produce equal hatch frequencies. We assume that the relative fecundity of infected females is *F* = 1 − *s*_f_ and that mating is random with respect to infection status. Assuming equal infection frequencies in males and females, adult infection frequency in generation *t*, denoted *p_t_,* changes between generations as follows:

[1] $$p_{t+1}=\;\frac{p_{t}F(1-\mu )}{1+p_{t}\left(F-1-s_{h}\right)+p_{t}^{2}s_{h}(1-\mu F)}\approx p_{t}F(1-\mu )\;\;\text{for}\;p_{t}\approx 0$$

(67). Notably, a *Wolbachia* infection will tend to increase when rare only if *F*(1 − μ) > 1, whether or not it causes CI. If *F*(1 − μ) < 1, then 0 is a stable equilibrium. The fecundity parameter *F* approximates more general fitness effects.

As demonstrated by Kreisner et al. (53), with sufficient positive fitness effects and CI (i.e., *F*(1 – μ) > 1, *s*_h_ > 0, and *Fμ* < 1), there is a unique stable equilibrium frequency between 0 and 1, namely:

[2] $${\hat{p}}_{s}=\;\frac{s_{h}+1-F+\sqrt{{\left(s_{h}+1-F\right)}^{2}+4s_{h}[F\left(1-\mu \right)-1](1-F\mu )}}{2s_{h}(1-F\mu )}.$$

For infections that do not cause CI (*s*_h_ = 0) but enhance fitness sufficiently that *F*(1 – μ) > 1, the stable equilibrium is

[3] $${\hat{p}}_{s}=1-\frac{\mu F}{F-1}$$

(3).

With perfect maternal transmission (μ = 0) but *F* < 1, *p* = 0 is a stable equilibrium infection frequency. As noted by Caspari and Watson (69), if the level of CI, as measured by *s*_h_ = 1 − *H*, exceeds the fitness cost of infection, as measured by *s*_f_ = 1 − *F*, 1 is also a stable equilibrium infection frequency, with an intermediate unstable equilibrium at

[4] $${\hat{p}}_{u}=s_{\text{f}}/s_{\text{h}}.$$

With imperfect maternal transmission, μ > 0, and *F*(1 − μ) < 1, *p* = 0 is a stable equilibrium frequency, as noted above. If *s*_h_ is sufficiently large and μ sufficiently small (see Eq. **4** in ref. 26), the additional stable and unstable polymorphic equilibria satisfy the same quadratic that produces Eq. **2**. The stable equilibrium infection frequency, ${\hat{p}}_{s}$, is given by Eq. **2**; and the unstable equilibrium is

[5] $${\hat{p}}_{u}=\;\frac{s_{h}+1-F-\sqrt{{\left(s_{h}+1-F\right)}^{2}+4s_{h}[F\left(1-\mu \right)-1](1-F\mu )}}{2s_{h}(1-F\mu )}.$$

For μ = 0 and *s*_h_ > *s*_f_, ${\hat{p}}_{u}\;$ in Eq. **5** reduces to Eq. **4**. We use this deterministic model and the finite-population stochastic generalization below to make our key points.

### Stochastic Effects of Finite Population Size.

As in the analysis of Turelli and Barton (51), we approximate the stochasticity induced by finite population size using a stochastic transition matrix, described below, analogous to a haploid Wright–Fisher model of genetic drift. These dynamics can be accurately approximated using standard diffusion theory, as shown by Jansen et al. (50) and illustrated below. The model uses an effective population size (70), which we denote by *N*. (Because *Wolbachia* are generally maternally transmitted, *N* is the effective number of females.) This finite-population stochasticity, modeled as binomial sampling, is superimposed on the deterministic dynamics described by Eq. **1**.

Assuming discrete generations and constant (effective) adult female population size *N*, let *I_t_* denote the number of *Wolbachia*-infected reproductive females in generation *t*, so that *p_t_* = *I_t_*/*N*. The stochastic transition matrix *Q* = (*q_ij_*) is defined as

[6] $$q_{ij}=P\left(I_{t}{}_{+1}=j|I_{t}=i\right),$$

(i.e., the probability of going from *i* to *j* infected females in one generation). We approximate these probabilities using two assumptions: 1) starting with the current adult (female) infection frequency, *p_t_* = *I_t_*/*N*, the infection frequency among viable gametes in the next generation is determined by the deterministic recursion Eq. **1**; and 2) the infection frequency in the next generation of *N* adult females is obtained from binomial sampling of this deterministic projection. These assumptions correspond to the usual Wright–Fisher approximation (70). Letting p* denote the expected frequency from Eq. **1** [i.e., (*p_t_*_+1_ | *p_t_* = *I_t_*/*N*)], the elements of *Q* are

[7] $$q_{ij}=\;\left(\begin{array}{c} N\\ j \end{array}\right){\left(p^{*}\right)}^{j}{\left(1\;–\;p^{*}\right)}^{N\;–\;j}.$$

We use this model to approximate both establishment probabilities in new host species and persistence times of *Wolbachia* infections within individual host species. When considering establishment probabilities, we simplify the analysis by assuming perfect maternal transmission in Eq. **1** so that establishment corresponds to reaching fixation at *p* = 1.

### An Epidemiological Model Describing CI Prevalence among Host Species.

Werren and Windsor (39) introduced an epidemiological model to understand the fraction of *Wolbachia*-infected insect species. After describing their model, using alternative notation, we generalize it to describe the interspecific frequency dynamics of CI-causing and non-CI-causing *Wolbachia*. Our epidemiological models treat species as individuals, which can be infected or uninfected. We assume that the global collection of potential host species can be approximated as a single “well-mixed” population. More realistic transmission models, describing networks of contact and preferential transmission associated with geographic or phylogenetic distances between hosts (e.g., see ref. 71), should be considered to evaluate the robustness of our qualitative conclusions.

Werren and Windsor’s (39) analysis corresponds to a susceptible-infected-susceptible (SIS) disease model with “mass action” transmission (see Eq. 2.44 in ref. 64) in which uninfected species are “susceptible” to new *Wolbachia* infections, whereas “infected” species are immune to additional infections, until those infections are lost, at which point the species again becomes susceptible. Let *I* denote the fraction of infected species and *U* = 1 − *I* denote the uninfected. The model has only two parameters: β, which describes the rate of transmission between host species (see Box 2.1 in ref. 64 for a derivation and interpretation), and γ, the loss rate (so that the average duration of a specific *Wolbachia* infection in a host species is 1/γ). The standard SIS model is

[8] $$\frac{dI}{dt}=\;\beta IU\;-\;\gamma I$$

Assuming β > γ, so that *I* increases when near 0, this equation has a unique stable equilibrium at $\hat{I}$ = 1 − (γ/β), as noted by Werren and Windsor (39).

SIS model Eq. **8** for disease prevalence within a host species is said to be “without demography” because it ignores births and deaths, assumes that the infection does not affect longevity, and assumes that the infection dynamics occur on a timescale shorter than individual host life spans. When applied to *Wolbachia* incidence across host species, Eq. **8** assumes that *Wolbachia* infections do not affect species longevity and that they are typically acquired and lost on a timescale faster than species durations. Given that many *Wolbachia* infections were acquired relatively recently and that most seem to be acquired by introgression or nonsexual horizontal transmission (see Table 2 in ref. 71; 32, 34, 72), our generalization of Eq. **8** will ignore potential *Wolbachia* effects on speciation and extinction rates of host species. We return to this in our *Discussion*.

We generalize Eq. **8** to consider both CI-causing and non-CI-causing *Wolbachia*. We assume, for simplicity, that each host species harbors only one *Wolbachia* infection, so that only uninfected species can become infected via direct (introgression) or indirect (nonsexual horizontal transmission) “contact” with an infected heterospecific host. Contrary to this assumption, some host species harbor multiple *Wolbachia* infections within individuals [e.g., the parasitic wasp *Nasonia vitripennis* (73), close relatives of *N*. *vitripennis* (74), the tephritid *Rhagoletis cerasi* (75), and various *Drosophila* species listed below]. Also, some host species have different *Wolbachia* in different geographic locations (e.g., *D. simulans*; refs. 10 and 76). Nevertheless, relatively few host species seem to harbor multiple distinct *Wolbachia* infections, at least among drosophilids. Of about 70 drosophilid species surveyed with generally intermediate or high *Wolbachia* prevalence (see discussions in refs. 2 and 30), double and alternative *Wolbachia* infections have been reported in only three: *D. simulans* (10, 77), *D. sechellia* (77), and *D. pandora* (78). Based on mitochondrial introgression in the three-species *D. yakuba* clade, we expect that some closely related host species may be polymorphic for very closely related *Wolbachia* variants because of recent introgression (55). For our purposes, such host species would be considered singly infected. Our broad-scale analysis ignores complications associated with multiple *Wolbachia* infections within individual host species.

We denote by *I* the fraction of potential host species infected with CI-causing *Wolbachia* and by *I*_0_ the fraction infected with non-CI-causing *Wolbachia*. By assumption, *U*, the fraction of uninfected potential host species, is *U* = 1 − *I* − *I*_0_. The transmission and loss rates associated with CI-causing and non–CI-causing *Wolbachia* are denoted (β, γ) and (β_0_, γ_0_), respectively. Finally, we assume that CI-causing *Wolbachia* infections are converted to non-CI-causing infections at rate *c*. Loss of CI can be caused by either loss of functional CI loci within a *Wolbachia* lineage (15, 29, 30) or by host suppression of the *Wolbachia* reproductive manipulation (5, 79). Although host modulation of CI is known (e.g., by *D. melanogaster*; compare refs. 5 and 6), complete loss of CI seems generally associated with the loss of functional CI-causing loci from the *Wolbachia* genome (15, 29, 30). For simplicity, we ignore the fact that CI-inducing loci can be acquired by horizontal transmission between *Wolbachia* lineages (15, 30) and assume that the loss of CI within *Wolbachia* lineages is permanent. Our extension of Eq. **8** is

[9a] $$\frac{dI}{dt}=\;\beta IU\;-\;\gamma I\;-cI\;\text{and}$$

[9b] $$\frac{dI_{0}}{dt}=\;\beta_{0}I_{0}U\;-\;\gamma_{0}I_{0}+cI.$$

This is analogous to the “complete cross-immunity” epidemiological model for the dynamics of two infections within a host species, as described by Eq. 4.1 in ref. 64.

**Fig. 1.**
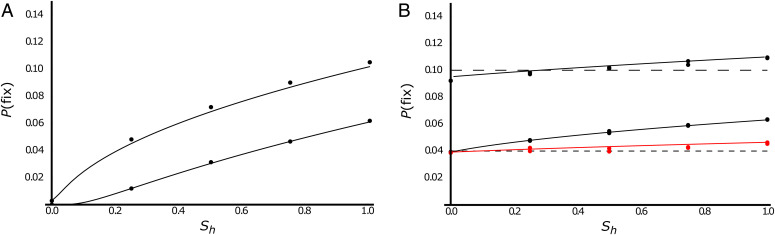
Effects of CI on fixation probabilities for initially rare deleterious (*A*) and mutualistic (*B*) *Wolbachia* infections with perfect maternal transmission (μ = 0). Both panels assume that a single infected female is introduced and plot the probability of fixation, denoted *P*(fix), as a function of the level of CI, with *s*_h_ = 1 − *H* denoting the proportional decrease of embryo viability caused by CI. The dots are estimates based on computer simulations; the solid lines are diffusion approximations (*Materials and Methods*). The effective population size is assumed to be 100 in *A*, whereas it is assumed to be 1,000 (black) or 5,000 (red) in *B*. (*A*), *F*, the relative fecundity of *Wolbachia*-infected females, is either 0.99 (upper line) or 0.95 (lower). (*B*), *F* = 1.05 (upper) or 1.02 (lower). The dotted lines in *B* provide the Haldane (83) approximation *P*(fix) ∼ 2(*F* − 1).

As Kriesner et al. (53) showed using Eq. **2**, CI-causing infections will generally have high equilibrium frequencies within their hosts. Hence, “contacts” between host species harboring CI-causing infections and uninfected heterospecifics are more likely to lead to *Wolbachia* transmission (i.e., we expect β > β_0_). This frequency-based argument applies to both beneficial and deleterious *Wolbachia*. However, for deleterious *Wolbachia*, we expect β ≫ β_0_, because only deleterious infections that cause CI are likely to become established in a new host after an initial low-frequency introduction (50). To successfully invade a new host, deleterious *Wolbachia* require drift to push them over the unstable equilibrium frequency described by Eq. **5**. As illustrated by Fig. 1, this will generally require a small effective population size. As illustrated below, we also expect CI-causing infections to persist longer in host species (i.e., γ < γ_0_; expected infection durations are 1/γ and 1/γ_0_), whether or not they are deleterious.

Assuming that *I* increases when near zero (i.e., β > γ + *c*), Eq. **9a** implies that

[10a] $$\hat{U}=(\gamma +c)/\beta .$$

Eq. **9b** implies that

[10b] $${\hat{I}}_{0}/\hat{I}=c/(\gamma_{0}\;–\;\beta_{0}\hat{U}).$$

Thus, ${\hat{I}}_{0}\hat{I}$ > 0 only if *c* > 0 and $\gamma_{0}$ − $\beta_{0}\hat{U}$ > 0. This model produces a unique equilibrium compatible with the empirical approximations that $\hat{U}\;\approx 1/2$ and $\hat{I}\;\approx \;{\hat{I}}_{0}$ among insect species. To see this, note that the constraints $\hat{I}=\;{\hat{I}}_{0}$ and $\hat{U}=1/2$ produce two equations for *c*. Both are satisfied if γ + γ_0_ = (β + β_0_)/2 (i.e., the sum of the loss rates is half the sum of the gain rates). The unique equilibrium, described by Eq. **10** with ${\hat{I}}_{0}\hat{I}$ > 0, is at least locally stable (*SI Appendix*). Our estimate that approximately half of the *Wolbachia* infections studied cause detectable CI may be an overestimate. As discussed below, CI-causing infections will generally have higher frequencies within host species, making them easier to detect in multispecies *Wolbachia* surveys.

Eq. **10b** implies that ${\hat{I}}_{0}$ > 0 only when *c* > 0 (i.e., non–CI-causing *Wolbachia* persist only because they are regularly produced by loss of functional CI-causing loci). Without this conversion process, our epidemiological analysis suggests that only CI-causing *Wolbachia* would occur. Because β /γ > β_0_ /γ_0_ and because two infections with complete cross-immunity cannot stably co-occur [the one with the higher ratio of transmission to loss rates, i.e., β/γ versus β_0_/γ_0,_ is expected to prevail (64)], our model suggests that non-CI-causing *Wolbachia* in nature should generally derive from CI-causing ancestors, a prediction that can tested with phylogenetic analyses. (This prediction remains valid if we generalize the model by allowing non-CI-causing *Wolbachia* to be converted to CI-causing by the transfer of CI-causing loci between *Wolbachia* lineages. This conversion process is exemplified by the *Wolbachia* in the three-species *D. yakuba* clade (55, 80) and more generally supported by the phylogenetic incongruence between *Wolbachia* “core” genomes, CI-causing loci, and the phage that contain them (81)).

## Results

Our conclusions are motivated by the idealized epidemiological model discussed above, which considers the movement of *Wolbachia* between host species, the loss of *Wolbachia* infections by host species, and the loss of CI within *Wolbachia* lineages. We next illustrate how CI both enhances transmission of *Wolbachia* between host species and the persistence of *Wolbachia* infections within host species. We then present old and new data supporting our central assumption that many *Wolbachia* infections in insect host species are young relative to the timescale of host speciation and extinction.

### Theoretical Results Concerning Establishment, Transmission, and Persistence.

We first quantify the effects of CI on the probability that a rare *Wolbachia* variant successfully invades a new host species, contrasting deleterious and mutualistic variants. CI dramatically improves invasion success for deleterious variants, as noted by Jansen et al. (50), but we show below that it only minimally aids invasion by mutualistic variants. For both deleterious and mutualistic variants, we argue that CI is favored in two ways. First, as illustrated by Kriesner et al. (53), CI-causing variants will generally be more abundant within host species than non-CI-causing variants and, hence, more likely to be introduced into new hosts by nonsexual horizontal transmission and introgression. Second, we use simulations to demonstrate that CI-causing *Wolbachia* are likely to persist longer within host lineages than non-CI-causing variants, because they can be maintained at high frequencies even if environmental changes make their effects on host fitness fluctuate between mutualistic and harmful.

### Establishment in New Host Species: Deleterious versus Beneficial *Wolbachia.*

As suggested by Hurst and McVean (24) and quantified by Jansen et al. (50), CI greatly increases the establishment probabilities for *Wolbachia* that satisfy *F*(1 − μ) < 1. Fig. 1*A* illustrates this effect, assuming perfect maternal transmission (μ = 0) with effective population size *N* = 100 and *F* = 0.99 or 0.95. (For simplicity, these simulations ignore imperfect maternal transmission so that *Wolbachia* “establishment” can be identified with fixation.) Without CI, fixation probabilities, denoted *P*(fix), for deleterious *Wolbachia* are negligible (0.003 for *F* = 0.99 and ≪10^−4^ for *F* = 0.95). Deleterious *Wolbachia* that induce CI produce an unstable threshold frequency (Eq. **5**), which, once exceeded, tends to produce a high stable infection frequency (Eq. **2**). However, because CI is effectively nonexistent at low *Wolbachia* frequencies (compare with Eq. **1**), random fluctuations of infection frequencies are essential to establishing deleterious *Wolbachia* from low-frequency introductions. In contrast, with *F*(1 − μ) > 1, Eq. **1** shows that infection frequencies tend to increase deterministically.

Bistability does not apply to non-CI-causing variants. Most *Wolbachia* that have been studied in natural populations show imperfect maternal transmission (e.g., see refs. 2, 62, and 82), thus we do not expect non-CI-causing variants to persist unless they are sufficiently mutualistic to satisfy *F*(1 − μ) > 1 (40). Assuming that many natural *Wolbachia* infections satisfy *F*(1 − μ) > 1, at least when they initially invade a host species, we can ask whether CI helps establish mutualistic *Wolbachia*. Based on estimates of imperfect maternal transmission rates that are typically on the order of a few percent (e.g., see refs. 26, 62, and 68), we focus on fitness increases of a few percent. Assuming *N* = 1,000, with *F* = 1.02 or 1.05, Fig. 1*B* shows that CI has a much smaller relative effect on invasion success for *Wolbachia* satisfying *F*(1 − μ) > 1 in comparison to variants with *F*(1 − μ) < 1 (Fig. 1*A*). As the level of CI changes from nonexistent (*H* = 1, so *s*_h_ = 0) to complete (*H* = 0, so *s*_h_ = 1), the probability of fixation remains close to the classic Haldane (83) approximation for the probability of fixation of a single-copy favorable mutation, namely 2(*F* − 1) (assuming perfect maternal transmission). For *F* = 1.02, our simulations show that the fixation probability for a new *Wolbachia* infection, introduced into a single female, increases from 0.04 with no CI (*s*_h_ = 0) to 0.06 for complete CI (*s*_h_ = 1). Although appreciable, this is minimal compared with the orders-of-magnitude effect seen when *F*(1 − μ) < 1 (Fig. 1*A*). Fig. 1 also shows that the probabilities estimated from simulations agree closely with a diffusion-based predictions (compare with ref. 50).

The effective population sizes in Fig. 1 *A* and *B* were chosen to produce similar values for the fixation probabilities, *P*(fix). For significantly deleterious *Wolbachia* infections (e.g., *F* = 0.95), the fixation probability plummets as *N* increases from 100 to 1,000. For *F* = 0.95 and *N* = 1000, *P*(fix) is only 0.002, even with complete CI (*s*_h_ = 1) [in contrast to *P*(fix) ≈ 0.036 and 0.049 for *F* = 1 and *F* = 1.01, respectively]. The effect of increasing *N* is far less dramatic for very weakly deleterious infections. For instance, with *F* = 0.99 and complete CI, *P*(fix) ≈ 0.024, which is approximately half the value, *P*(fix) ≈ 0.050, obtained with *F* = 1.01. Hence, even though CI dramatically enhances establishment probabilities for significantly deleterious CI-causing infections (e.g., those with *F* ≤ 0.95), such infections are very unlikely to establish in new hosts after rare introductions, except in very small populations. Given that CI does little to enhance establishment probabilities for mutualistic *Wolbachia* in new host species (Fig. 1*B*), what role might it play in their transmission across species and maintenance within species? We present two alternatives, both of which apply whether or not the CI-causing *Wolbachia* are mutualistic.

### Transmission to New Host Species: Frequency within Host Species.

Kriesner et al. (see Eq. **5** and Fig. 9 in ref. 53) showed that a relatively small amount of CI significantly increases the equilibrium population frequencies of *Wolbachia* satisfying *F*(1 − μ) > 0. Eq. **5** of in the report by Kriesner et al. (53) implies that with *s*_h_ as small as 0.22 (*H* ≤ 0.78), the minimum stable equilibrium frequency is at least 0.8 for μ ≤ 0.05. In contrast, the best-studied non-CI-causing *Wolbachia* infections rarely achieve population frequencies above 0.4 [e.g., *w*Au in *D. simulans* (40) and *w*Mau in *D. mauritiana* (30)]. Hence, *Wolbachia* that produce CI are generally more common within species and more likely to be spread by horizontal transmission. This “mass action” effect can involve either introgression or nonsexual horizontal transmission: the more common an infection is within host species, the more likely it is to be transferred. For mutualistic *Wolbachia*, this effect on intrapopulation prevalence will generally exceed the small effect of CI on establishment probabilities illustrated in Fig. 1*B*.

### Transmission to New Host Species: Persistence of Infections within Host Species.

**Fig. 2.**
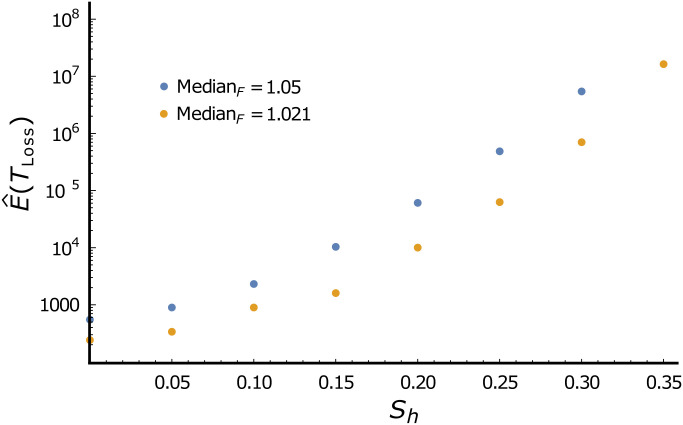
Effect of CI on the expected persistence times of *Wolbachia* infections when fitness effects fluctuate. As expected, persistence times increase with higher median *Wolbachia* fitness effects (blue versus gold) and more intense CI (increasing *s*_h_). The simulations assume that the relative fecundity, *F*, of infected females fluctuates across generations as independent, identically distributed log-normal random variables with CV*_F_* = 0.4. This corresponds to extreme variation in *F*. With median(*F*) = 1.05, the 0.025 and 0.975 percentiles are 0.49 and 2.23, respectively; with median(*F*) = 1.021, the corresponding values are 0.48 and 2.17. The effective female population size is 1,000 and maternal *Wolbachia* transmission is imperfect with μ = 0.02, so that eventual loss is certain. The estimates presented are the average over 25 replicate simulations. Persistence times are approximately exponentially distributed, so the SE for each estimate is approximately one-fifth of the estimated mean.

The proposed effect of CI on persistence times of *Wolbachia* infections within host species is condition dependent. The idea is that if an established, initially advantageous, CI-causing *Wolbachia* infection becomes deleterious, CI can maintain it at a high stable equilibrium frequency, as expressed by Eq. **2**. This bistability, with alternative stable equilibria at 0 and near 1, is central to applications of fitness-decreasing *Wolbachia* transinfections to disease control (48, 51). If the current frequency in a population is above the unstable equilibrium, described by Eq. **5**, we expect the infection to stably persist. As predicted, field data indicate that introduced, clearly deleterious *Wolbachia* transinfections in *Ae. aegypti* have remained near fixation for over a decade after establishment through systematic introductions, based on repeated large releases (49, 84). In contrast, without CI, if an infection becomes deleterious, its frequency will deterministically decline. We illustrate the potential consequences of fluctuating fitness effects by considering persistence times of *Wolbachia* infections in finite populations in which conditions fluctuate so that the infection is sometimes advantageous, with *F*(1 − μ) > 1, and sometime deleterious, with *F*(1 − μ) < 1. Without CI (*s*_h_ = 0 in Fig. 2), infections are lost relatively rapidly because of the deterministic push toward 0 when *F*(1 − μ) < 1. We have no empirical guidance to choose plausible parameters; but Fig. 2, which provides simulation-based estimates of expected persistence times, denoted $\hat{E}(T_{\mathrm{Loss}})$, measured in generations, illustrates the principle that CI-causing *Wolbachia* are likely to persist much longer.

The dramatic effect displayed in Fig. 2 of CI intensity on expected *Wolbachia* persistence times results from assuming extreme temporal fluctuations in fitness effects. We know too little about *Wolbachia* fitness effects in nature to make useful quantitative predictions (but see ref. 61 for data supporting condition-dependent *Wolbachia* effects). Nevertheless, the qualitative conclusion is robust: CI-induced bistability surely promotes *Wolbachia* persistence within host lineages if fitness effects fluctuate between mutualistic and deleterious through time. Increased persistence obviously enhances transmission to new hosts. A comparable effect can be produced by fluctuating levels of maternal transmission.

### Variation of *Wolbachia* Frequencies in Space and Time.

Our epidemiological analysis assumes that *Wolbachia* infections are lost and gained on a shorter timescale than speciation and extinction of hosts. Recent acquisition of current *Wolbachia* infections closely related to *w*Ri (“*w*Ri-like”), initially described in *D. simulans* (4), has been demonstrated for several *Drosophila* species (32). Within about 15,000 y, *w*Ri-like infections have been acquired by at least eight species, including *D. simulans* and *D. ananassae*. These hosts span the *D. melanogaster* species group, which diverged about 25 Mya (85, 86). Cooper et al. (55) presented comparable data concerning *Wolbachia* closely related to *w*Mel, initially found in *D. melanogaster* (52). We have expanded the Cooper et al. (55) analyses to include at least 15 drosophilid hosts, including *D. melanogaster* and *Zaprionus tsacasi*, which diverged over 40 Mya (86). As with the hosts analyzed by Cooper et al. (55), these more distantly related drosophilids all acquired *w*Mel-like infections over approximately 80,000 y.

**Fig. 3.**
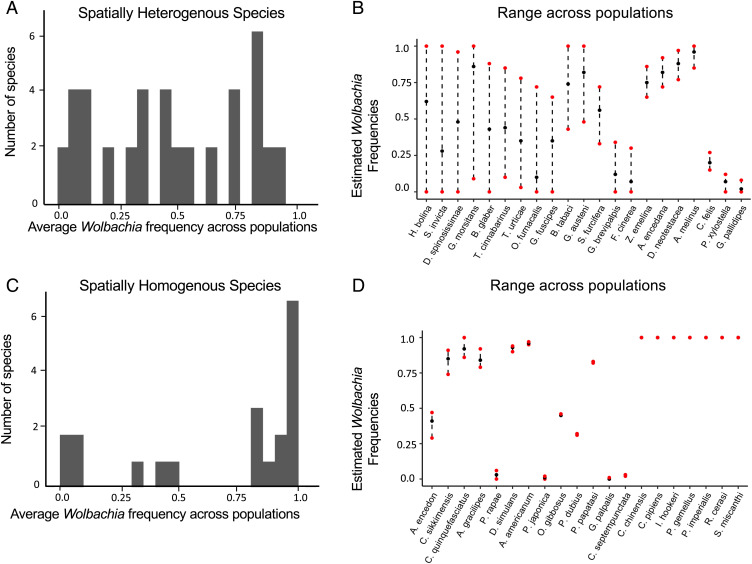
Estimated *Wolbachia* frequencies from 41 host species with detectable *Wolbachia* infections in which at least 30 individuals were sampled within each of at least two populations. (*A*) The mean infection frequencies (arithmetic means over populations, not weighted by sample sizes) for the 21 species showing statistically significant (*P* < 0.05) spatial heterogeneity in frequencies. (*B*) For the 21 species from *A*, abbreviated species names are listed and the ranges of estimated intraspecific infection frequencies, ordered from largest to smallest ranges. The dark blue dots in *B* show the unweighted arithmetic mean frequencies across populations (i.e., the values plotted in *A*). (*C*) The mean infection frequencies from the 20 infected species displaying no statistically significant (*P* > 0.05) spatial heterogeneity in frequencies. (*D*) The names and ranges of intraspecific frequency estimates for the 20 species from *C*.

Within *D. simulans*, *w*Ri replaced *w*Au in eastern Australia within 20 y (40). Spatial spread of *Wolbachia* variants have been observed in *D. simulans* (25, 40), *Laodelphax striatellus* (87), and *Rhagoletis cerasi* (88). These data suggest that *Wolbachia* infections may regularly turn over within host species. In Fig. 3 and *SI Appendix*, Table S1 and Fig. S1, we summarize additional data indicating that spatial and temporal *Wolbachia* spread may be common among arthropods. Our survey began with data compiled by Weinert et al. (1). We focused on 51 species for which there were at least two geographically distinct samples, each including at least 30 individuals. The *Wolbachia* incidence among these 51 species (i.e., the fraction of species in which *Wolbachia* was detected) was 0.80 (*n* = 41 of 51). This incidence estimate is obviously biased upward by the fact that researchers are more likely to publish *Wolbachia* frequency data from multiple populations if *Wolbachia* has been detected. For instance, an additional 133 species in the Weinert et al. (1) collection had samples of individual populations with *n* ≥ 30 (*SI Appendix,* Table S2). Of them, 65 of 133 (49%) had detectable *Wolbachia* infections, consistent with the overall incidence estimate reported in in ref. 1.

Fig. 3 summarizes the infection frequency data from *SI Appendix*, Table S1. Of the 41 infected species, approximately half (*n* = 21 of 41) show statistically significant (*P* < 0.05) heterogeneity of infection frequencies among populations. Fig. 3*A* shows the mean infection frequencies for these 21 “heterogeneous” species, and Fig. 3*B* shows the range of intraspecific frequency estimates. Of these 21 species, eight show maximum interpopulation differences in *Wolbachia* frequency estimates of at least 0.7 (Fig. 3*B*). For six of them, the maximum frequency estimate is at least 0.85, suggesting that their *Wolbachia* infections typically produce CI [*w*Bol1 in *Hypolimnas bolina* causes both CI and male-killing (89)] and may be spreading (or contracting) spatially. More strikingly, 4 of the 41 infected species show at least one population in which *Wolbachia* was not detected and another in which the estimated infection frequency was at least 0.88 (*Solenopsis invicta*, *Diplolepsis spinosissimae*, *Balloniscus glaber*, *H. bolina*). Hence, for at least 4 of the 41 *Wolbachia*-infected species surveyed, an infection seems to be spatially spreading or contracting. Four other species, in which interpopulation frequency estimates differ by at least 0.7 (*Glossina morsitans*, *Ostrinia furnacalis*, *Tetranychus cinnabarinus*, *T. urticae*), are also plausible candidate hosts for spatial spread or retreat, further indicating regular turnover of *Wolbachia* infections. Moreover, among the 21 infected species with significant spatial heterogeneity, 10 have at least one population in which *Wolbachia* was not detected (Fig. 3*B*).

Fig. 3*C* presents average *Wolbachia* frequencies from the 20 infected species that show no statistically significant (*P* > 0.05) spatial heterogeneity in frequencies, and Fig. 3*D* shows the frequency ranges. The difference between Fig. 3 *A* and *C* in the number of species showing intermediate average frequencies reflects the extreme spatial heterogeneity shown by several species in Fig. 3*B*. Note that some species show very low estimated *Wolbachia* frequencies; for instance, *Propylaea japonica*, *Coccinella septempunctata*, and *G. palpalis* all have maximum estimated intrapopulation frequencies of, at most, 0.03. These species may have nonheritable, somatic *Wolbachia* infections (90). Thus, the data in *SI Appendix*, Table S1 may underestimate the frequency with which *Wolbachia* infections show spatial heterogeneity and plausible spread.

Fig. 3 shows that spatial variation in *Wolbachia* frequencies is common. However, the pattern of spatial variation indicated by these data is obfuscated by sparse and variable annotation of sampling sites, taken at different times, from the primary studies. Among the 20 species with partially or fully annotated sampling locations, three were identified as having a clear visual pattern of clinically varying infection frequencies: *S. invicta*, *T. urticae*, and *B. glaber* (*SI Appendix*, Fig. S1). The *S. invicta* data suggest two distinct *Wolbachia* introductions are associated with this species’ recent appearance in North America (91), reminiscent of the separate introductions of *w*Ri into northern and southern populations of *Drosophila simulans* in eastern Australia. The available spatial and temporal survey data seem consistent with the hypothesis that *Wolbachia* infections are regularly in flux and spatial sweeps may well be relatively common. However, spatially varying frequencies may also represent relatively stable clines associated with spatially varying fitness effects or maternal transmission, as seen with *w*Mel in Australian *D. melanogaster* populations (53, 62). Frequency variation in space and time is also known for *Wolbachia* that cannot cause CI, either because they lack functional CI-causing loci [e.g., *w*Au (81)] or because asexual host reproduction precludes CI (e.g., see refs. 92 and 93). Some of these examples may represent introduction and loss, as with *w*Au in eastern Australia (40), but some may be transients associated with fluctuations in effects on host fitness or transmission efficiency. Comparable spatial and temporal frequency variation is observed for non-CI-causing, non-*Wolbachia* endosymbionts that are condition-dependent mutualists (e.g., see refs. 94 and 95). As discussed below, data from non-CI-causing facultative endosymbionts, whether *Wolbachia* or not, serve as controls for our predictions concerning clade-selection advantages of CI.

## Discussion

Why is *Wolbachia*-based CI so common even though natural selection does not favor, or even preserve, CI among *Wolbachia* variants within a host population? We argue that the key is clade selection based on preferential transmission of CI-causing variants to new host species and longer persistence of CI-causing variants within host lineages. Chronograms estimated from genomic data (32, 55), observed spatial spread (25, 40, 87, 88), and spatially varying frequencies within host species (Fig. 3) indicate that *Wolbachia* infections are often gained and lost by host species significantly faster than typical speciation and extinction times. This turnover provides an opportunity for differential proliferation of *Wolbachia* variants across potential hosts. Building on the work of Hurst and McVean (24), we argue that CI is common because higher intraspecific frequencies make CI-causing *Wolbachia* lineages more likely to spread to new hosts than non-CI lineages, and once established in new hosts, CI-causing *Wolbachia* are likely to persist much longer (Fig. 2). Both the transmission and persistence advantages apply to both mutualistic and deleterious *Wolbachia*. In contrast, the clade-selection argument of Hurst and McVean (24) rested on a proposed invasion-probability advantage for CI-causing *Wolbachia* that is appreciable only for deleterious variants (Fig. 1).

How might our hypothesis be tested? First, we predict that CI-causing *Wolbachia* infections should generally have higher frequencies within host populations than non-CI-causing *Wolbachia* infections. High frequencies for CI-causing infections follow directly from simple models (53), but the comparison with non-CI-causing *Wolbachia* rests on the testable assumption that facultative mutualisms tend to produce lower population frequencies because their positive fitness effects are context dependent and likely to be less intense than the frequency-dependent advantage produced by strong CI. A related prediction is that *Wolbachia* variants that cause strong CI (e.g., *w*Ri in *D. simulans*) should show significantly less temporal and spatial population-frequency variations than *Wolbachia* variants that cause little CI (e.g., *w*Mel in *D. melanogaster*) or no CI (e.g., *w*Au in *D. simulans*). Additional spatial and temporal *Wolbachia* surveys are needed that control for CI levels. Even for host taxa that are difficult to rear in the laboratory, the existence of CI can now be plausibly inferred from the presence of apparently functional CI-causing loci (14, 15); and in nature, one can compare egg-hatch frequencies for embryos produced by co-occurring infected versus uninfected females. Third, CI-causing *Wolbachia* should persist longer than non-CI-causing *Wolbachia* in host species, corresponding to older average ages (longer branches) in *Wolbachia* chronograms. Finally, non-CI-causing *Wolbachia* should generally appear at the tips of *Wolbachia* phylogenies, as relatively recent descendants of CI-causing variants, assuming that the pairs of loci that produce CI are more easily lost than gained. This is analogous to the phylogenetic placement of parthenogenetic eukaryotic lineages (96). Phylogenetic and functional analyses of 71 *Wolbachia* genomes by Martinez et al. (15) broadly support this prediction.

After discovering bidirectional CI among a geographical patchwork of *Culex pipiens* populations, Laven (97) conjectured that CI may be important in producing new insect species. The existence of CI between spatially isolated *Nasonia* species (98), the apparent role of CI in the reinforcement of reproductive isolation between two closely related *Drosophila* species (99), and in the reproductive isolation between “semispecies” of the *D. paulistorum* clade (100) all seem consistent with a potential role for *Wolbachia* in speciation. However, the paucity of very closely related *Drosophila* species showing high levels of intrinsic postzygotic isolation (101), despite the pervasiveness of *Wolbachia* infections among *Drosophila* species, and the young age of many current *Wolbachia* infections, including those in *C. pipiens* (102), make it increasingly implausible that *Wolbachia* contribute frequently to the origin of species (compare refs. 54 and 103). Convincing evidence of widespread *Wolbachia* effects on speciation or extinction rates will require incidence data and phylogenetic estimates for hundreds of host species (104). We expect that *Wolbachia* effects on the birth and death of host species will be much smaller than the effects of CI on the persistence of *Wolbachia* within host species and transmission rates of *Wolbachia* between host species.

Our idealized epidemiological model dichotomizes *Wolbachia* variants into those that do or do not cause CI. For simplicity, it assumes that the CI-causing variants can lose their functional CI-causing loci but does not allow for reacquisition of such loci or the accumulation of multiple *Wolbachia* variants within hosts, both of which are known to occur (30, 77). These complications are obviously relevant to understanding the diversity of *Wolbachia* infections in nature. However, our basic arguments about the prevalence of CI-causing *Wolbachia*, which focus on establishment in new hosts and the frequency and persistence of CI-causing *Wolbachia* variants (relative to non-CI-causing variants) within host species, do not depend on the epidemiological details of *Wolbachia* evolution.

Our mathematical analyses simplistically assume that all *Wolbachia* variants can invade all potential hosts. In fact, the *Wolbachia* within insect orders show significant phylogenetic affinity (15), presumably reflecting coevolution between *Wolbachia* and their hosts. Similarly, we know that host individuals can harbor multiple *Wolbachia* lineages, a fact ignored by our idealized treatment. More realistic analyses will require additional data, but our qualitative conclusions about clade selection advantages associated with CI-producing variants seem robust. Our models of preferential spread among host species make testable predictions that are consistent with existing data. Simple calculations show that CI is not favored by natural selection acting among mutually compatible *Wolbachia* variants within host species (22, 23, 28). Hence, differential proliferation of CI-causing lineages across their broad range of potential arthropod hosts provides a plausible explanation for this phenotype that is pervasive among what may be the most successful group of facultative intracellular symbionts.

## Materials and Methods

### Establishment Probabilities: Simulations and Fiffusion Approximations.

Our simulations begin with a single infected female in a female population of effective size *N*. For simplicity, we assume perfect maternal transmission (μ = 0). We simulated population frequencies using Eqs. **1** and **7** until the infection was fixed (*I_t_* = *N*) or lost (*I_t_* = 0), and calculated the fraction of trials that ended with fixation. The numerical results were compared with an analytical diffusion approximation, described below, and the classic Haldane (83) approximation for the probability of fixation of a single favorable mutation, namely 2(*F* − 1).

To produce the solid lines in Fig. 1, we used a diffusion approximation to describe the stochastic dynamics produced by the transition matrix, Eq. **7** (compare with ref. 50). This approximation characterizes the model’s behavior by the infinitesimal mean, denoted *m*(*p*), and the infinitesimal variance, denoted *v*(*p*), which approximate E(Δ*p_t_* | *p_t_* = *p*) and Var(Δ*p_t_* | *p_t_* = *p*), respectively (105). For simplicity, we assume perfect maternal transmission (μ = 0) so that *Wolbachia* establishment corresponds to fixation at *p* = 1. We use the numerator of the deterministic recursion Δ*p_t_* = *p_t_*_+ 1_ − *p_t_* derived from Eq. **1** to approximate the infinitesimal mean, *m*(*p*), and binomial sampling variance to approximate the infinitesimal variance; that is,

[11a] $$m\left(p\right)=s_{\text{h}}p\left(1-p\right)(p-\hat{p}),\;\text{with}\;\hat{p}=s_{\text{f}}/s_{\text{h}},\;\text{and}$$

[11b] v(p) =p(1−p)/N.

For *p* ≈ 0, *m*(*p*) ≈ −*ps*_f_. For an initial frequency of *p*_0_, the diffusion approximation implies that the probability of *Wolbachia* fixation is

[12a] $$\text{P}\left(\text{fix}\;|{\;\text{p}}_{0}\right)=\int_{0}^{p_{0}}G\left(x\right)dx/\int_{0}^{1}G\left(x\right)dx,\;\text{with}$$

[12b] $$\text{G}\left(\text{x}\right)=\text{Exp}\left(-2\int_{}^{x}\left[\frac{m\left(y\right)}{v\left(y\right)}\right]dy\right)=\text{kExp}\left[\text{xN}\left({\text{2s}}_{\text{f}}-{\text{s}}_{\text{h}}\text{x}\right)\right],$$

where *k* is an arbitrary constant, and *m*(*y*) and *v*(*y*) are given by Eq. **11**. To produce Fig. 1, we used Mathematica 13.0.1 to numerically evaluate Eq. **12**, using *p*_0_ = 1/*N*. *P*(fix | *p*_0_) can be expressed in terms of incomplete error functions, but they also require numerical evaluation [except to produce the Haldane (83) approximation for fixation probabilities].

### Simulations to Approximate Infection Durations under Fluctuating Conditions.

We simulated fluctuating *Wolbachia* effects on host fitness by assuming that the fitness parameter *F* in Eq. **1** is a random variable. For simplicity, we assumed that each generation *F* is chosen independently from a lognormal distribution (i.e., *F* = e*^X^*, where *X* is a normal random variable with mean μ_X_ and variance σ^2^). This implies that *F* has median *m* = $e^{\mu_{X}}$ and squared coefficient of variation (CV^2^) = Var(*F*)/[E(*F*)]^2^ = $e^{\sigma^{2}}$− 1. Thus, to produce a particular median, *m*, and CV for *F*, we set μ_X_ = ln(*m*) and σ^2^ = ln(CV^2^ + 1). We assume fixed female effective population size, *N*, and fixed levels of CI, parameterized by *s*_h_ = 1 − *H*. To insure that the *Wolbachia* infection is ultimately lost, we assume imperfect maternal transmission (i.e., μ > 0 in Eq. **1**). Starting with an intermediate infection frequency, arbitrarily chosen at *p*_0_ = 0.4 (using *p*_0_ = 0.2 makes no appreciable difference), we simulated population infection frequencies according to the transition matrix Eq. **7** with stochastically varying *F* in Eq. **1** until the infection is lost. We present the mean persistence time as a function of the level of CI in Fig. 2.

### *Wolbachia* Frequency Variation in Space.

We used a subset of the data from the Weinert et al. (1) meta-analysis on endosymbiont incidence to identify and analyze relatively large (*n* ≥ 30) intraspecific population samples screened for *Wolbachia* in at least two separate locations. For each population, the Weinert et al. (1) database provided a taxonomic identifier, the number of individuals screened for *Wolbachia*, and the number infected. We validated and added collection-site locations for these population samples by referring to the publications cited in *SI Appendix*, Table S1. For 51 arthropod species, there were at least two *n* ≥ 30 population samples (a total of 330 population samples). We categorized the infections in these 51 species as homogeneous or heterogeneous, using the χ^2^ test with a significance level of *P* ≤ 0.05 (*SI Appendix*, Table S1). To identify potential spatial spread, we estimated latitude and longitude of collection sites using the R package *ggmap*. *Wolbachia* population infection data with latitude and longitude were plotted in R using the package *ggplot2* and Google Maps API. Trends in infection frequency covarying with geography were assessed visually as well as quantitatively by the Mann-Whitney *U* statistical test. We excluded from the geographic analysis samples whose locations were only broadly described (e.g., country of origin). For only three species did we observe clear spatial clines in infection frequency (*SI Appendix*, Fig. S1).

## Supplementary Material

Supplementary File

## Data Availability

All study data are included in the article and/or supporting information. Previously published data were used for this work (1).
