# Supplementary material for: Why Wolbachia-induced cytoplasmic incompatibility is so common
Source: Proc Natl Acad Sci U S A. 2022 Nov 7;119(47):e2211637119. doi: 10.1073/pnas.2211637119 (PMC9704703; doi:10.1073/pnas.2211637119)
Supplement: Supplementary File [file pnas.2211637119.sapp.pdf]

Supporting information

Why *Wolbachia*-induced cytoplasmic incompatibility is so common

Michael Turelli, Andrew Katznelson, and Paul S. Ginsberg

Appendix S1: Local stability of the epidemiological equilibrium

We show that the unique equilibrium described by Eq. 10 for our epidemiological model Eq. 9 is locally stable. The Jacobian for Eq. 9 evaluated at equilibrium Eq. 10 can be written as

$$J = \begin{pmatrix} -\beta\hat{I} & -\beta\hat{I} \\ -\beta_0\hat{I}_0 + c & -\beta_0\hat{I}_0 - c[\hat{I}/\hat{I}_0] \end{pmatrix} = \begin{pmatrix} -a & -a \\ -b + c & -b - cd \end{pmatrix}, \quad (1)$$

with $a > 0$, $b > 0$, $c > 0$ and $d > 0$, where c is the loss-of-CI parameter in Eq. 9 and the composite parameters a, b and d are defined by (1). The characteristic equation produced by (1) is

$$\lambda^2 + A\lambda + B = 0, \quad (2)$$

with $A = a + b + cd > 0$, $B = ac(1 + d) > 0$, and a, b, c and d as defined in (1). The corresponding eigenvalues determining local stability are

$$\lambda = \frac{1}{2}\left(-A \pm \sqrt{A^2 - 4B}\right). \quad (3)$$

There are two cases to consider depending on the sign of $A^2 - 4B$. For both, the eigenvalues in (3) have negative real parts and hence that the equilibrium of Eq. 9 described by Eq. 10 is locally stable. If $A^2 - 4B > 0$, both eigenvalues are real and obviously negative because $A > 0$ and $B > 0$. If $A^2 - 4B < 0$, the eigenvalues are complex, but $\mathrm{Re}(\lambda_i) = -A/2 < 0$.

Appendix S2: *Wolbachia* incidence and geographic variation

Table S1 summarizes data extracted from references in Weinert et al. (2015) for 51 species in which in which at least 30 individuals were sampled from each of at least two populations (with references to the original data sources). Weinert et al. (2015) reported data from an

additional 133 species with a single population sample of at least 30 individuals (Table S2). Of those 133 species, significantly fewer showed detectable *Wolbachia* infections than the 51 species reported in Table S1 (65/133 versus 41/51, $\chi^2_1 = 13.7$, $P < 0.001$), consistent with the expectation that Table S1 species represent a sample biased upward with respect to *Wolbachia* incidence. However, among the 65 infected species in Table S2, 20/65 have estimated *Wolbachia* frequency ≥ 0.85, consistent with the fraction of species in Table S1 having high-frequency populations (8/41, $\chi^2_1 = 1.1$, $P > 0.29$). Similarly, the fraction of those 65 infected species in Table S2 with very low infection frequencies (≤ 0.03), namely 3/65, is consistent with the fraction of species with low-frequency infections from Table S1 (3/41, $\chi^2_1 = 0.02$, $P > 0.5$). These observations are consistent with the hypothesis that the *Wolbachia* infections summarized in Table S1 are typical of those found in nature.

Table S1. *Wolbachia* frequencies estimated from 41 species in which at least 30 individuals were sampled from each of at least two populations (curated data from Weinert et al. 2015, with references to the original data sources).

Order	Genus	Species	Number of Populations	Mean of Population Frequencies	Maximum	Minimum	Standard Deviation	Variation Among Populations	References
Araneae	*Oedothorax*	*gibbosus*	2	0.45	0.46	0.45	0	Homogeneous	[1]
Araneae	*Misumenops*	*tricuspidatus*	2	Uninfected					[2, 3]
Coleoptera	*Adalia*	*bipunctata*	2	Uninfected					[4]
Coleoptera	*Adalia*	*decempunctata*	2	Uninfected					[4]
Coleoptera	*Anisosticta*	*novemdecimpunctata*	2	Uninfected					[4]
Coleoptera	*Myrrha*	*octodecimguttata*	2	Uninfected					[4]
Coleoptera	*Coccinella*	*septempunctata*	2	0.03	0.03	0.02	0	Homogeneous	[4]
Coleoptera	*Subcoccinella*	*vigintiquatuorpunctata*	2	Uninfected					[4]
Coleoptera	*Propylaea*	*japonica*	3	0.01	0.02	0	0.01	Homogeneous	[2, 3, 5]
Coleoptera	*Curculio*	*sikkimensis*	3	0.85	0.91	0.74	0.09	Homogeneous	[6]
Coleoptera	*Callosobruchus*	*chinensis*	6	1	1	1	0	Homogeneous	[7]
Diptera	*Glossina*	*austeni*	6	0.82	1	0.48	0.25	Heterogeneous	[8, 9]
Diptera	*Glossina*	*brevipalpis*	3	0.12	0.34	0	0.19	Heterogeneous	[8, 9]
Diptera	*Glossina*	*fuscipes*	6	0.35	0.65	0	0.3	Heterogeneous	[8, 9, 10, 11]
Diptera	*Glossina*	*morsitans*	9	0.86	1	0.09	0.29	Heterogeneous	[9, 12]
Diptera	*Drosophila*	*neotestacea*	15	0.88	0.97	0.77	0.08	Heterogeneous	[13]
Diptera	*Glossina*	*pallidipes*	10	0.02	0.08	0	0.03	Heterogeneous	[8, 9]
Diptera	*Glossina*	*longipennis*	2	Uninfected					[5 , 8]
Diptera	*Phlebotomus*	*papatasi*	2	0.83	0.83	0.82	0.01	Homogeneous	[14]
Diptera	*Culex*	*pipiens*	3	1	1	1	0	Homogeneous	[15, 16, 17]
Diptera	*Rhagoletis*	*cerasi*	4	1	1	1	0	Homogeneous	[18]
Diptera	*Glossina*	*palpalis*	6	0	0.01	0	0	Homogeneous	[9]
Diptera	*Drosophila*	*simulans*	9	0.93	0.94	0.9	0.01	Homogeneous	[19, 20, 21]
Diptera	*Culex*	*quinquefasciatus*	16	0.92	1	0.86	0.04	Homogeneous	[15, 22]
Hemiptera	*Sogatella*	*furcifera*	5	0.56	0.72	0.33	0.15	Heterogeneous	[5, 23, 24]
Hemiptera	*Bemisia*	*tabaci*	6	0.74	1	0.43	0.23	Heterogeneous	[25, 26, 27, 28, 29]
Hemiptera	*Sitobion*	*miscanthi*	6	1	1	1	0	Homogeneous	[30]
Hemiptera	*Acyrthosiphon*	*pisum*	7	Uninfected					[31, 32, 33]

Hymenoptera	*Formica*	*cinerea*	8	0.07	0.3	0	0.1	Heterogeneous	[34]
Hymenoptera	*Solenopsis*	*invicta*	30	0.10	0.75	0	0.17	Heterogeneous	[35, 36, 37, 38, 39]
Hymenoptera	*Aphytis*	*melinus*	20	0.96	1	0.85	0.04	Heterogeneous	[40]
Hymenoptera	*Diplolepis*	*spinosissimae*	2	0.48	0.96	0	0.68	Heterogeneous	[41]
Hymenoptera	*Pachycrepoideus*	*dubius*	2	0.32	0.32	0.31	0.01	Homogeneous	[42]
Hymenoptera	*Pegoscapus*	*gemellus*	2	1	1	1	0	Homogeneous	[43, 44]
Hymenoptera	*Ixodiphagus*	*hookeri*	2	1	1	1	0	Homogeneous	[45]
Hymenoptera	*Pleistodontes*	*imperialis*	2	1	1	1	0	Homogeneous	[44, 46]
Hymenoptera	*Anoplolepis*	*gracilipes*	3	0.84	0.92	0.79	0.07	Homogeneous	[47]
Isopoda	*Balloniscus*	*glaber*	5	0.43	0.88	0	0.36	Heterogeneous	[48]
Ixodida	*Amblyomma*	*americanum*	2	0.04	0.05	0.03	0.01	Homogeneous	[49]
Lepidoptera	*Hypolimnas*	*bolina*	16	0.62	1	0	0.34	Heterogeneous	[50, 51, 52, 53, 54]
Lepidoptera	*Zizina*	*emelina*	2	0.75	0.86	0.65	0.15	Heterogeneous	[55]
Lepidoptera	*Acraea*	*encedana*	2	0.82	0.92	0.72	0.15	Heterogeneous	[56]
Lepidoptera	*Ostrinia*	*furnacalis*	39	0.1	0.72	0	0.13	Heterogeneous	[57, 58]
Lepidoptera	*Plutella*	*xylostella*	3	0.07	0.12	0	0.06	Heterogeneous	[59]
Lepidoptera	*Acraea*	*encedon*	4	0.41	0.47	0.29	0.08	Homogeneous	[60]
Lepidoptera	*Pieris*	*rapae*	4	0.03	0.06	0	0.02	Homogeneous	[61]
Mesostigmata	*Dermanyssus*	*gallinae*	2	Uninfected					[62]
Prostigmata	*Tetranychus*	*cinnabarinus*	5	0.44	0.85	0.1	0.33	Heterogeneous	[63, 64]
Prostigmata	*Tetranychus*	*urticae*	17	0.35	0.78	0.03	0.21	Heterogeneous	[63, 65, 67]
Prostigmata	*Panonychus*	*citri*	2	Uninfected					[63, 67]
Siphonaptera	*Ctenocephalides*	*felis*	3	0.2	0.27	0.15	0.06	Heterogeneous	[68, 69]

Table S2. *Wolbachia* frequencies estimated from 133 species in which at least 30 individuals were sampled from a single population (curated data from Weinert et al. 2015, with references to the original data sources).

Order	Genus	Species	N	Infection Frequency	References
Araneae	*Araneus*	*mitificus*	30	0	[2]
Araneae	*Araneus*	*ventricosus*	30	0.66	[2]
Araneae	*Argiope*	*amoena*	30	0	[2]
Araneae	*Eriovixia*	*cavaleriei*	30	0.26	[2]
Araneae	*Larinia*	*argiopiformis*	30	0.4	[2]
Araneae	*Larinioides*	*cornutus*	30	0	[2]
Araneae	*Clubiona*	*japonicola*	30	0	[2]
Araneae	*Erigone*	*prominens*	30	0	[2]
Araneae	*Hylyphantes*	*graminicola*	30	0	[2]
Araneae	*Neriene*	*limbatinella*	30	0	[2]
Araneae	*Neriene*	*radiata*	30	0	[2]
Araneae	*Ummeliata*	*insecticeps*	30	0	[2]
Araneae	*Pardosa*	*astrigera*	30	0	[2]
Araneae	*Pardosa*	*laura*	30	0	[2]
Araneae	*Pardosa*	*pseudoannulata*	30	0	[2]
Araneae	*Pirata*	*piraticus*	30	0	[2]
Araneae	*Pirata*	*piratoides*	30	0	[2]
Araneae	*Pirata*	*subpiraticus*	30	0	[2]
Araneae	*Pirata*	*tenuisetaceus*	30	0	[2]
Araneae	*Nephila*	*clavata*	30	0.63	[2]
Araneae	*Oxyopes*	*sertalus*	30	0.73	[2]
Araneae	*Pholcus*	*crypticolens*	30	0.56	[2]
Araneae	*Marpissa*	*magister*	30	0	[2]
Araneae	*Plexippus*	*paykulli*	30	0	[2]
Araneae	*Tetragnatha*	*praedonia*	30	0	[2]
Araneae	*Tetragnatha*	*squamata*	30	0	[2]
Araneae	*Coleosoma*	*octomaculatum*	30	0.13	[2]
Araneae	*Parasteatoda*	*tepidariorum*	30	0	[2]
Araneae	*Thomisus*	*labefactus*	30	0	[2]
Coleoptera	*Aulacophora*	*indica*	70	0	[26]
Coleoptera	*Monolepta*	*signata*	33	0	[5]
Coleoptera	*Anatis*	*ocellata*	163	0	[4]
Coleoptera	*Aphidecta*	*obliterata*	44	0	[4]
Coleoptera	*Calvia*	*quatuordecimguttata*	57	0.01	[4]
Coleoptera	*Coccidula*	*rufa*	49	0.57	[4]
Coleoptera	*Coccinella*	*hieroglyphica*	83	0	[4]
Coleoptera	*Coccinella*	*miranda*	146	0	[4]

Coleoptera	*Exochomus*	*quadripustulatus*	95	0	[4]
Coleoptera	*Halyzia*	*sedecimguttata*	260	0.01	[4]
Coleoptera	*Harmonia*	*quadripunctata*	33	0	[4]
Coleoptera	*Micraspis*	*discolor*	131	0	[5]
Coleoptera	*Myzia*	*oblongoguttata*	85	0	[4]
Coleoptera	*Rhyzobius*	*litura*	70	0.61	[4]
Coleoptera	*Tytthaspis*	*hexadecpunctata*	53	0	[4]
Coleoptera	*Pityogenes*	*chalcographus*	30	0.5	[70]
Collembola	*Onychiurus*	*sinensis*	37	0	[71]
Diptera	*Orseolia*	*oryzae*	38	0.18	[5]
Diptera	*Aedes*	*albopictus*	143	0.91	[72]
Diptera	*Culex*	*tarsalis*	30	0	[15]
Diptera	*Drosophila*	*dasycnemia*	56	0.21	[73]
Diptera	*Drosophila*	*innubila*	2861	0.34	[74]
Diptera	*Drosophila*	*orientacea*	33	0.9	[13]
Diptera	*Glossina*	*tachinoides*	93	0	[8]
Diptera	*Lutzomyia*	*trapidoi*	30	0.53	[75]
Diptera	*Bactrocera*	*tau*	44	0.06	[26]
Hemiptera	*Leptocorisa*	*oratorius*	49	0	[5]
Hemiptera	*Amphorophora*	*rubi*	109	0	[32]
Hemiptera	*Aphis*	*sarothamni*	42	0	[32]
Hemiptera	*Microlophium*	*carnosum*	101	0	[32]
Hemiptera	*Cofana*	*spectra*	33	0.03	[5]
Hemiptera	*Empoascanara*	*alami*	37	0.02	[5]
Hemiptera	*Nephotettix*	*nigropictus*	46	0.06	[5]
Hemiptera	*Nephotettix*	*virescens*	292	0.11	[5]
Hemiptera	*Recilia*	*dorsalis*	116	0.05	[5]
Hemiptera	*Thaia*	*oryzivora*	45	0	[5]
Hemiptera	*Laodelphax*	*striatellus*	51	1	[23]
Hemiptera	*Nilaparvata*	*lugens*	237	0.16	[5]
Hemiptera	*Cyrtorhinus*	*lividipennis*	221	0.23	[5]
Hemiptera	*Halticus*	*minutus*	129	0.37	[26]
Hemiptera	*Macrolophus*	*pygmaeus*	40	1	[76]
Hymenoptera	*Bombus*	*terrestris*	46	1	[77]
Hymenoptera	*Andricus*	*caputmedusae*	38	0	[78]
Hymenoptera	*Andricus*	*coriarius*	30	0	[78]
Hymenoptera	*Andricus*	*coronatus*	35	0	[78]
Hymenoptera	*Andricus*	*lucidus*	35	0	[78]
Hymenoptera	*Andricus*	*panteli*	31	0	[78]
Hymenoptera	*Cynips*	*quercus*	31	0	[78]
Hymenoptera	*Synergus*	*gallaepomiformis*	34	0.85	[78]
Hymenoptera	*Trichopria*	*cf_drosophilae*	30	0.9	[42]
Hymenoptera	*Pnigalio*	*soemius*	107	0.03	[79]

Hymenoptera	*Acromyrmex*	*echinatior*	383	0.41	[80]
Hymenoptera	*Acromyrmex*	*octospinosus*	1198	0.45	[80]
Hymenoptera	*Atta*	*colombica*	202	0.6	[80]
Hymenoptera	*Atta*	*sexdens*	342	0.69	[80]
Hymenoptera	*Trissolcus*	*festivae*	37	0.97	[81]
Hymenoptera	*Trissolcus*	*flavipes*	38	0.92	[81]
Hymenoptera	*Trissolcus*	*grandis*	46	0.91	[81]
Hymenoptera	*Trissolcus*	*rufiventris*	48	1	[81]
Hymenoptera	*Trissolcus*	*semistriatus*	70	0	[81]
Hymenoptera	*Trichogramma*	*ostriniae*	101	1	[82]
Isopoda	*Armadillidium*	*vulgare*	32	0.06	[76]
Isopoda	*Balloniscus*	*sellowii*	38	0.13	[48]
Ixodida	*Dermacentor*	*marginatus*	61	0	[83]
Ixodida	*Ixodes*	*ricinus*	799	0	[84]
Lepidoptera	*Chilo*	*partellus*	30	0	[85]
Lepidoptera	*Cnaphalocrocis*	*medinalis*	86	0.48	[5]
Lepidoptera	*Ostrinia*	*nubilalis*	62	0	[86]
Lepidoptera	*Phyllonorycter*	*blancardella*	60	1	[87]
Lepidoptera	*Lycaeides*	*melissa*	30	0.03	[88]
Lepidoptera	*Talicada*	*nyseus*	60	0.5	[89]
Lepidoptera	*Zizeeria*	*maha*	81	0.96	[90]
Lepidoptera	*Acraea*	*acerata*	71	1	[91]
Lepidoptera	*Acraea*	*aganice*	60	0	[91]
Lepidoptera	*Acraea*	*consanguinea*	39	0	[91]
Lepidoptera	*Acraea*	*eponina*	39	0.12	[91]
Lepidoptera	*Acraea*	*lycoa*	30	0	[91]
Lepidoptera	*Acraea*	*peneleos*	60	0.03	[91]
Lepidoptera	*Acraea*	*perenna*	30	0	[91]
Lepidoptera	*Acraea*	*pseudoegina*	35	0	[91]
Lepidoptera	*Acraea*	*servona*	44	0	[91]
Lepidoptera	*Ariadne*	*merione*	30	0	[88]
Lepidoptera	*Danaus*	*chrysippus*	30	0	[88]
Lepidoptera	*Danaus*	*genutia*	30	0	[88]
Lepidoptera	*Euploea*	*core*	30	0	[88]
Lepidoptera	*Neonympha*	*mitchellii*	180	0.11	[92]
Lepidoptera	*Polygonia*	*c-album*	85	0.96	[93]
Lepidoptera	*Speyeria*	*zerene*	66	0.03	[94]
Lepidoptera	*Tirumala*	*limniace*	30	0	[88]
Lepidoptera	*Papilio*	*demoleus*	30	0	[88]
Lepidoptera	*Papilio*	*polymnestor*	60	0.5	[88]
Lepidoptera	*Catopsilia*	*pyranthe*	30	0	[88]
Lepidoptera	*Eurema*	*hecabe*	72	1	[95]
Odonata	*Agriocnemis*	*femina*	69	0.08	[96]

Odonata	*Agriocnemis*	*pygmaea*	235	0	[97]
Prostigmata	*Panonychus*	*mori*	52	0.09	[67]
Prostigmata	*Panonychus*	*osmanthi*	44	0	[67]
Prostigmata	*Tetranychus*	*pueraricola*	38	0.47	[67]
Prostigmata	*Tetranychus*	*truncates*	40	0.87	[63]
Prostigmata	*Tetranychus*	*turkestani*	40	0	[63]
Prostigmata	*Tetranychus*	*viennensis*	40	1	[63]
Siphonaptera	*Ceratophyllus*	*garei*	47	0.04	[95]
Siphonaptera	*Echidnophaga*	*gallinacea*	101	0.23	[68]
Siphonaptera	*Tunga*	*penetrans*	58	1	[98]

Fig. S1 shows spatial gradients in *Wolbachia* infection frequencies for three species: *Solenopsis invicta* (red fire ant), *Tetranychus urticae* (red spider mite) and *Balloniscus glaber* (isopod).

Fig. S1. Apparent spatial gradients in *Wolbachia* infection frequencies for three host species.

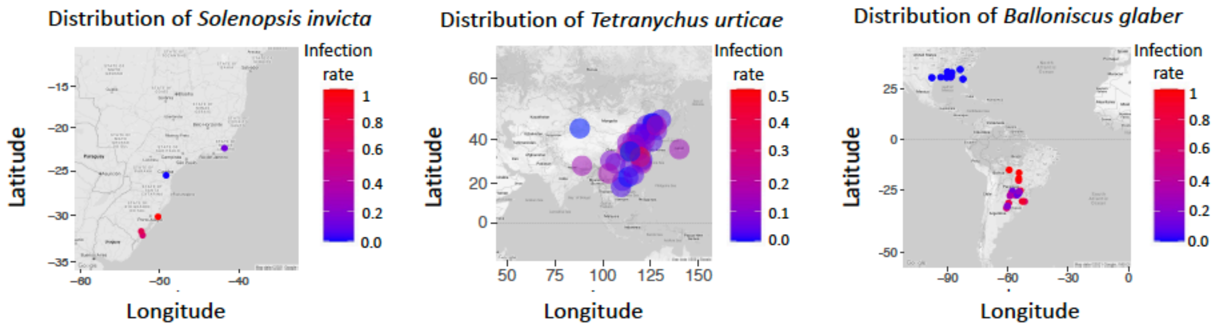

SI References

1. B. Vanthournout, J. Swaegers, F. Hendrickx, Spiders do not escape reproductive manipulations by *Wolbachia*. *BMC Evol. Biol.* **11**, 15 (2011).
2. Z. Wang, C. Deng, Y. Yun, C. Jian, Y. Peng, Molecular detection and the phylogenetics of *Wolbachia* in Chinese spiders (Araneae). *J. Arachnology* **38**, 237–241 (2010).
3. Y. Yun, Y. Peng, F. X. Liu, C. Lei, *Wolbachia* Screening in spiders and assessment of horizontal transmission between predator and prey. *Neotropical Entomology* **40**, 164–169 (2011).
4. L. A. Weinert, M. C. Tinsley, M. Temperley, F. M. Jiggins, Are we underestimating the diversity and incidence of insect bacterial symbionts? A case study in ladybird beetles. *Biology Letters* **3**, 678–681 (2007).
5. P. Kittayapong, W. Jamnongluk, A. Thipaksorn, J. R. Milne, C. Sindhusake, *Wolbachia* infection complexity among insects in the tropical rice-field community. *Mol. Ecol.* **12**, 1049–1060 (2003).
6. H. Toju, T. Fukatsu, Diversity and infection prevalence of endosymbionts in natural populations of the chestnut weevil: relevance of local climate and host plants. *Mol. Ecol.* **20**, 853–868 (2011).
7. N. Kondo, M. Shimada, T. Fukatsu, High prevalence of *Wolbachia* in the azuki bean beetle *Callosobruchus chinensis* (Coleoptera, Bruchidae). *Zoological Science* **16**, 955–962 (1999).
8. Q. Cheng et al., Tissue distribution and prevalence of *Wolbachia* infections in tsetse flies, *Glossina* spp. *Med. Vet. Entomology* **14**, 44–50 (2000).
9. V. Doudoumis et al., Detection and characterization of *Wolbachia* infections in laboratory and natural populations of different species of tsetse flies (genus *Glossina*). *BMC Microbiology* **12 (Suppl. 1)**, S3 (2012).
10. Alam, U. et al., Implications for trypanosome transmission dynamics in *Glossina fuscipes fuscipes* in Uganda. *Appl. Environ. Micro*. 78, 4627–4637 (2012).
11. R. E. Symula et al., *Wolbachia* association with the tsetse fly, *Glossina fuscipes fuscipes*, reveals high levels of genetic diversity and complex evolutionary dynamics. *BMC Evol. Biol.* **13**, 31 (2013).
12. D. I. Schneider, K. I. Garschall, A. G. Parker, A. M. M. Abd-Alla, W. J. Miller, Global *Wolbachia* prevalence, titer fluctuations and their potential of causing cytoplasmic incompatibilities in tsetse flies and hybrids of *Glossina morsitans* subgroup species. *J. Invert. Path.* **112 Suppl**, S104–115 (2013).
13. J. Jaenike, J. K. Stahlhut, L. M. Boelio, R. L. Unckless, Association between *Wolbachia* and *Spiroplasma* within *Drosophila neotestacea*: an emerging symbiotic mutualism? *Mol. Ecol.* **19**, 414–425 (2010).
14. M. Benlarbi, P. D. Ready, Host-specific *Wolbachia* strains in widespread populations of *Phlebotomus perniciosus* and *P. papatasi* (Diptera: Psychodidae), and prospects for driving genes into these vectors of Leishmania. *Bull. Entomol. Res*. **93**, 383–391 (2003).
15. J. L. Rasgon, T. W. Scott, An initial survey for *Wolbachia* (Rickettsiales: Rickettsiaceae) infections in selected California mosquitoes (Diptera: Culicidae). *J. Med. Entomol*. **41**, 255–257 (2004).
16. C. M. Atyame et al., Multiple *Wolbachia* determinants control the evolution of cytoplasmic incompatibilities in *Culex pipiens* mosquito populations. *Mol. Ecol*. **20**, 286–298 (2011).

17. O. Duron, M. Raymond, M. Weill, Many compatible *Wolbachia* strains coexist within natural populations of *Culex pipiens* mosquito. *Heredity* **106**, 986–993 (2011).
18. W. Arthofer et al., Hidden *Wolbachia* diversity in field populations of the European cherry fruit fly, *Rhagoletis cerasi* (Diptera, Tephritidae). *Mol. Ecol*. **18**, 3816–3830 (2009).
19. C. H. Gorham, Q. Q. Fang, L. A. Durden, *Wolbachia* endosymbionts in fleas (Siphonaptera). *J. Parasitology* **89**, 283–289 (2003).
20. A. R. Weeks, M. Turelli, W. R. Harcombe, K. T. Reynolds, A. A. Hoffmann, From parasite to mutualist: rapid evolution of *Wolbachia* in natural populations of *Drosophila*. *PLoS Biology* **5**, e114 (2007).
21. L. B. Carrington, J. R. Lipkowitz, A. A. Hoffmann, M. Turelli, A re-examination of *Wolbachia*-induced cytoplasmic incompatibility in California *Drosophila simulans*. *PLoS One* **6**, e22565 (2011).
22. I. P. Sunish, R. Rajendran, R. Paramasivan, K. J. Dhananjeyan, B. K. Tyagi, *Wolbachia* endobacteria in a natural population of *Culex quinquefasciatus* from filariasis endemic villages of south India and its phylogenetic implication. *Tropical Biomedicine* **28**, 569–576 (2011).
23. H. Noda, Y. Koizumi, Q. Zhang, Q., K. J. Deng, Infection density of *Wolbachia* and incompatibility level in two planthopper species, *Laodelphax striatellus* and *Sogatella furcifera*. *Insect Biochem*. *Mol. Biol*. **31**, 727–737 (2001).
24. X.-F. Zhang, D.-X. Zhao, X.-Y. Hong, *Cardinium*-the leading factor of cytoplasmic incompatibility in the planthopper *Sogatella furcifera* doubly infected with *Wolbachia* and *Cardinium*. *Environ. Entomol*. **41**, 833–840 (2012).
25. Y. C. Hung, C. H. Wang, C. C. Ko, Studies on the secondary endosymbionts of *Bemisia tabaci* (Gennadius) (Hemiptera: Aleyrodidae) in Taiwan. *Formosan Entomologist* **25**, 145–157 (2005).
26. S. Sintupachee, J. R. Milne, S. Poonchaisri, V. Baimai, P. Kittayapong, Closely related *Wolbachia* strains within the pumpkin arthropod community and the potential for horizontal transmission via the plant. *Micro. Ecol*. **51**, 294–301 (2006).
27. M. Z. Ahmed et al., Genetic distinctions among the Mediterranean and Chinese populations of *Bemisia tabaci* Q biotype and their endosymbiont *Wolbachia* populations. *J. Appl. Entomol*. **133**, 733–741 (2009).
28. M. Z. Ahmed, S.-X. Ren, N. S. Mandour, J. M. Greeff, B.-L. Qiu, Prevalence of *Wolbachia* supergroups A and B in *Bemisia tabaci* (Hemiptera: Aleyrodidae) and some of its natural enemies. *J. Econ. Entomol*. **103**, 1848–1859 (2010).
29. D. Chu et al., Further insights into the strange role of bacterial endosymbionts in whitefly, *Bemisia tabaci*: comparison of secondary symbionts from biotypes B and Q in China. *Bull. Entomol. Research* **101**, 477–486 (2011).
30. Z. Wang, Z.-R. Shen, Y. Song, H.-Y. Liu, Z.-X. Li, Distribution and diversity of *Wolbachia* in different populations of the wheat aphid *Sitobion miscanthi* (Hemiptera: Aphididae) in China. *European J. Entomology* **106**, 49–55 (2009).
31. T. Tsuchida, R. Koga, H. Shibao, T. Matsumoto, T. Fukatsu, Diversity and geographic distribution of secondary endosymbiotic bacteria in natural populations of the pea aphid, *Acyrthosiphon pisum*. *Mol. Ecol*. **11**, 2123–2135 (2002).
32. S. Haynes et al., Diversity of bacteria associated with natural aphid populations. *Appl. Environ. Micro.* **69**, 7216–7223 (2003).

33. J. A. Russell et al., Uncovering symbiont-driven genetic diversity across North American pea aphids. *Mol. Ecol.* **22**, 2045–2059 (2013).
34. A. Sirviö, P. Pamilo, Multiple endosymbionts in populations of the ant *Formica cinerea*. *BMC Evol. Biol.*, **10**, 335 (2010). .
35. D. D. Shoemaker, K. G. Ross, L. Keller, E. L. Vargo, J. H. Werren, *Wolbachia* infections in native and introduced populations of fire ants (*Solenopsis* spp.). *Insect Mol. Biol.* **9**, 661–673 (2000).
36. D. D. Shoemaker et al., Distribution and prevalence of *Wolbachia* infections in native populations of the fire ant *Solenopsis invicta* (Hymenoptera: Formicidae). *Environ. Entomol*. **32**, 1329–1336 (2003).
37. M. E. Ahrens, D. Shoemaker, Evolutionary history of *Wolbachia* infections in the fire ant *Solenopsis invicta*. *BMC Evol. Biol*. **5**, 35 (2005).
38. A. M. Bouwma, M. E. Ahrens, C. J. DeHeer, D. Shoemaker, Distribution and prevalence of *Wolbachia* in introduced populations of the fire ant *Solenopsis invicta*. *Insect Mol. Biol*. **15**, 89–93 (2006).
39. A. M. Bouwma, D. Shoemaker, *Wolbachia* w*Sinvicta*A infections in natural populations of the fire ant *Solenopsis invicta*: testing for phenotypic effects. *J. Insect Sci*. **11**, 11 (2011).
40. C. J. Vasquez, R. Stouthamer, G. Jeong, J. G. Morse, Discovery of a CI-inducing *Wolbachia* and its associated fitness costs in the biological control agent *Aphytis melinus* DeBach (Hymenoptera: Aphelinidae). *Biological Control* **58**, 192–198 (2011).
41. O. Plantard, J. Y. Rasplus, G. Mondor, I. Le Clainche, M. Solignac, Distribution and phylogeny of *Wolbachia* inducing thelytoky in Rhoditini and "Aylacini" (Hymenoptera: Cynipidae). *Insect Mol. Biol*. **8**, 185–191 (1999).
42. F. Vavre, F. Fleury, J. Varaldi, P. Fouillet, M. Boulétreau, Infection polymorphism and cytoplasmic incompatibility in Hymenoptera-*Wolbachia* associations. *Heredity* **88**, 361–365 (2002).
43. D. D. Shoemaker et al., The distribution of *Wolbachia* in fig wasps: correlations with host phylogeny, ecology and population structure. *Proc. Roy. Soc. Lond. B* **269**, 2257–2267 (2002).
44. M. Z. Ahmed, O. F. C. Greyvenstein, C. Erasmus, J. J. Welch, J. M. Greeff, Consistently high incidence of *Wolbachia* in global fig wasp communities. *Ecol. Entomol*. **38**, 147–154 (2013).
45. O. Plantard et al., Detection of *Wolbachia* in the tick *Ixodes ricinus* is due to the presence of the Hymenoptera endoparasitoid *Ixodiphagus hookeri*. *PLoS One* **7**, e30692 (2012).
46. E. R. Haine, J. M. Cook, Convergent incidences of *Wolbachia* infection in fig wasp communities from two continents. *Proc. Roy. Soc. Lond. B* **272**, 421–429 (2005).
47. A. Sebastien, M. A. M. Gruber, P. J. Lester, Prevalence and genetic diversity of three bacterial endosymbionts (*Wolbachia*, *Arsenophonus*, and *Rhizobiales*) associated with the invasive yellow crazy ant (*Anoplolepis gracilipes*). *Insectes Sociaux* **59**, 33–40 (2012).
48. M. P. Almerão et al., First record of *Wolbachia* in South American terrestrial isopods: prevalence and diversity in two species of *Balloniscus* (Crustacea, Oniscidea). *Genet. Mol. Biol.* **35**, 980–989 (2012).
49. X. Zhang, D. E. Norris, J. L. Rasgon, Distribution and molecular characterization of *Wolbachia* endosymbionts and filarial nematodes in Maryland populations of the lone star tick (*Amblyomma americanum*). *FEMS Microbiology Ecology* **77**, 50–56 (2011).

50. E. A. Dyson, M. K. Kamath, G. D. D. Hurst, *Wolbachia* infection associated with all-female broods in *Hypolimnas bolina* (Lepidoptera: Nymphalidae): evidence for horizontal transmission of a butterfly male killer. *Heredity* **88**, 166–171 (2002).
51. E. A. Dyson, G. D. D. Hurst, Persistence of an extreme sex-ratio bias in a natural population. *Proc. Natl. Acad. Sci. U.S.A.* **101**, 6520–6523 (2004).
52. S. Charlat et al., Prevalence and penetrance variation of male-killing *Wolbachia* across Indo-Pacific populations of the butterfly *Hypolimnas bolina*. *Mol. Ecol.* **14**, 3525–3530 (2005).
53. S. Charlat et al., Competing selfish genetic elements in the butterfly *Hypolimnas bolina*. *Current Biology* **16**, 2453–2458 (2006).
54. W. Mitsuhashi, H. Ikeda, M. Muraji, Fifty-year trend towards suppression of *Wolbachia*-induced male-killing by its butterfly host, *Hypolimnas bolina*. *J. Insect Sci.* **11**, 92 (2011).
55. Y. Sakamoto, N. Hirai, T. Tanikawa, M. Yago, M. Ishii, Infection by two strains of *Wolbachia* and sex ratio distortion in a population of the endangered butterfly *Zizina emelina* (Lepidoptera: Lycaenidae) in Northern Osaka Prefecture, Central Japan. *Ann. Entomol. Soc. America* **104**, 483–487 (2011).
56. S. S. M, Hassan, E. Idris, M. E. N. Majerus, Male-killer dynamics in the tropical butterfly, *Acraea encedana* (Lepidoptera: Nymphalidae). *Insect Science* **20**, 717–722 (2013).
57. D. Kageyama, G. Nishimura, S. Hoshizaki, Y. Ishikawa, Feminizing *Wolbachia* in an insect, *Ostrinia furnacalis* (Lepidoptera: Crambidae). *Heredity* **88**, 444–449 (2002).
58. J. Li, Z.-Y. Wang, D. Bourguet, K.-L. He, *Wolbachia* infection in populations of *Ostrinia furnacalis*: diversity, prevalence, phylogeny and evidence for horizontal transmission. *J. Integrative Agriculture* **12**, 283–295 (2013).
59. A. M. Delgado, J. M. Cook, Effects of a sex-ratio distorting endosymbiont on mtDNA variation in a global insect pest. *BMC Evol. Biol.* **9**, 49 (2009).
60. S. S. M, Hassan, E. Idris, M. E. N. Majerus, Morph ratio dynamics under male-killer invasion: The case of the tropical butterfly *Acraea encedon* (Lepidoptera: Nymphalidae). *J. Tropical Lepidoptera Research* **23**, 14–21 (2013).
61. Y. Tagami, K. Miura, Distribution and prevalence of *Wolbachia* in Japanese populations of Lepidoptera. *Insect Mol. Biol.* **13**, 359–364 (2004).
62. C. J. De Luna, C. V. Moro, J. H. Guy, L. Zenner, O. A. E. Sparagano, Endosymbiotic bacteria living inside the poultry red mite (*Dermanyssus gallinae*). Exp. And Appl. Acarology 48, 105–113 (2009).
63. X.-L. Chen, R.-R. Xie, G.-Q. Li, X.-Y. Hong, Simultaneous detection of endosymbionts *Wolbachia* and *Cardinium* in spider mites (Acari: Tetranychidae) by multiplex PCR. *Intl. J. Acarology* **35**, 397–403 (2009).
64. R.-R. Xie, L.-L. Zhou, Z.-J. Zhao, X.-Y. Hong. Male age influences the strength of *Cardinium*-induced cytoplasmic incompatibility expression in the carmine spider mite *Tetranychus cinnabarinus*. *Appl. Entomol. and Zoology* **45**, 417–423 (2010).
65. M.-Z. Yu, K.-J. Zhang, X.-F. Xue, X.-Y. Hong, Effects of *Wolbachia* on mtDNA variation and evolution in natural populations of *Tetranychus urticae* Koch. *Insect Mol. Biol.* **20**, 311–321 (2011).
66. H.-H. Su et al., Effects of *Wolbachia* on rDNA-ITS2 variation and evolution in natural populations of *Tetranychus urticae* Koch. *Syst. and Appl. Acarology* **17**, 45–52 (2012).

67. T. Gotoh, H. Noda, X.-Y. Hong, *Wolbachia* distribution and cytoplasmic incompatibility based on a survey of 42 spider mite species (Acari: Tetranychidae) in Japan. *Heredity* **91**, 208–216 (2003).
68. C. H. Gorham, Q. Q. Fang, L. A. Durden, *Wolbachia* endosymbionts in fleas (Siphonaptera). *J. Parasitology* **89**, 283–289 (2003).
69. J.-M. Rolain, M. Franc, B. Davoust, D. Raoult, Molecular detection of *Bartonella quintana*, *B. koehlerae*, *B. henselae*, B. *clarridgeiae*, *Rickettsia felis*, and *Wolbachia pipientis* in cat fleas, France. *Emerging Infectious Diseases* **9**, 339–342 (2003).
70. A. Wolfgang, R. Markus, A. Dimitrios, S. Christian, Evidence for low-titre infections in insect symbiosis: *Wolbachia* in the bark beetle *Pityogenes chalcographus* (Coleoptera, Scolytinae). *Environ. Micro.* **11**, 1923–1933 (2009).
71. F. Frati, I. Negri, P. P. Fanciulli, M. Pellecchia, R. Dallai, Ultrastructural and molecular identification of a new Rickettsia endosymbiont in the springtail *Onychiurus sinensis* (Hexapoda, Collembola). *J. Invert. Path*. **93**, 150–156 (2006).
72. A. L. Albuquerque, T. Magalhães, C. F. J. Ayres, High prevalence and lack of diversity of *Wolbachia pipientis* in *Aedes albopictus* populations from Northeast Brazil. *Memórias Do Instituto Oswaldo Cruz* **106**, 773–776 (2011).
73. G. M. Bennett, N. A. Pantoja, P. M. O'Grady, Diversity and phylogenetic relationships of *Wolbachia* in *Drosophila* and other native Hawaiian insects. *Fly* **6**, 1–11 (2012).
74. R. L. Unckless, J. Jaenike, Maintenance of a male-killing *Wolbachia* in *Drosophila innubila* by male-killing dependent and male-killing independent mechanisms. *Evolution* **66**, 678-689 (2012).
75. J. Azpurua, D. De La Cruz, A. Valderama, D. Windsor, Lutzomyia sand fly diversity and rates of infection by *Wolbachia* and an exotic Leishmania species on Barro Colorado Island, Panama. *PLoS Negl. Trop. Dis*. **4**, e627 (2010).
76. T. Machtelinckx et al., *Wolbachia* induces strong cytoplasmic incompatibility in the predatory bug *Macrolophus pygmaeus*. *Insect Mol. Biol*. **18**, 373–381 (2009).
77. S. E. Evison et al., Pervasiveness of parasites in pollinators. *PloS One* **7**, e30641 (2012).
78. A. Rokas, R. J. Atkinson, J.-L. Nieves-Aldrey, S. A. West, G. N. Stone, The incidence and diversity of *Wolbachia* in gallwasps (Hymenoptera; Cynipidae) on oak. *Mol. Ecol.* **11**, 1815–1829 (2002).
79. M. Gebiola, J. Gómez-Zurita, M. M. Monti, P. Navone, U. Bernardo, Integration of molecular, ecological, morphological and endosymbiont data for species delimitation within the *Pnigalio soemius* complex (Hymenoptera: Eulophidae). *Mol. Ecol.* **21**, 1190–1208 (2012).
80. S. Van Borm, T. Wenseleers, J. Billen, J. J. Boomsma, *Wolbachia* in leafcutter ants: a widespread symbiont that may induce male killing or incompatible matings. *J. Evol. Biol.* **14**, 805–814 (2001).
81. N. Guz, E. Kocak, A. E. Akpinar, M. O. Gurkan, A. N. Kilincer, *Wolbachia* infection in *Trissolcus* species (Hymenoptera: Scelionidae). *European J. Entomology* **109**, 169–174 (2012).
82. Y. Song, Z. R. Shen, Z. Wang, H. Y. Liu, Triple infection of *Wolbachia* in *Trichogramma ostriniae* (Hymenoptera: Trichogrammatidae). *Acta Entomologica Sinica* **52**, 445–452 (2009).

83. S. Hornok, et al., Molecular identification of *Anaplasma marginale* and rickettsial endosymbionts in blood-sucking flies (Diptera : Tabanidae, Muscidae) and hard ticks (Acari : Ixodidae). *Veterinary Parasitology* **154**, 354–359 (2008).
84. R. Václav, M. Ficová, P. Prokop, T. Betáková, Associations between coinfection prevalence of *Borrelia lusitaniae*, Anaplasma sp., and Rickettsia sp. in hard ticks feeding on reptile hosts. *Microbial Ecology* **61**, 245–253 (2011).
85. P. Pillay, et al., "Effects of *Wolbachia* infection on the interactions between *Eldana saccharina* and its insect parasitoids" In *Proceedings of the Annual Congress-South African Sugar Technologists' Association* (South African Sugar Technologists' Association, 2011), pp. 292–297.
86. D. Kageyama et al., *Wolbachia* infection and an all-female trait in *Ostrinia orientalis* and *Ostrinia zaguliaevi*. *Entomologia Experimentalis et Applicata* **111**, 79–83 (2004).
87. W. Kaiser, E. Huguet, J. Casas, C. Commin, D. Giron, Plant green-island phenotype induced by leaf-miners is mediated by bacterial symbionts. *Proc. Roy. Soc. Lond. B* **277**, 2311–2319 (2010).
88. C. C. Nice, Z. Gompert, M. L. Forister, J. A. Fordyce, An unseen foe in arthropod conservation efforts: The case of *Wolbachia* infections in the Karner blue butterfly. *Biological Conservation* **142**, 3137–3146 (2009).
89. K. Ankola, D. Brueckner, H. P. Puttaraju, *Wolbachia* endosymbiont infection in two Indian butterflies and female-biased sex ratio in the Red Pierrot, *Talicada nyseus*. *J. Biosciences* **36**, 845–850 (2011).
90. T. Sumi, K. Miura, T. Miyatake, No seasonal trend in infection of the pale grass blue butterfly, *Zizeeria maha* (Lepidoptera: Lycaenidae), by *Wolbachia*. Appl. Entomol. and Zool. **48**, 35–38 (2013).
91. F. M. Jiggins, J. K. Bentley, M. E. Majerus, G. D. Hurst, How many species are infected with Wolbachia? Cryptic sex ratio distorters revealed to be common by intensive sampling. *Proc. Roy. Soc. Lond. B* **268**, 1123–1126 (2001).
92. C. Hamm et al., *Wolbachia* infection and Lepidoptera of conservation concern. *J. Insect Science* **14**, 6 (2014).
93. U. Kodandaramaiah, E. Weingartner, N. Janz, L. Dalén, S. Nylin, Population structure in relation to host-plant ecology and *Wolbachia* infestation in the comma butterfly. *J. Evol. Biol*. **24**, 2173–2185 (2011).
94. A. McHugh et al., A molecular phylogenetic analysis of *Speyeria* and its implications for the management of the threatened *Speyeria zerene hippolyta*. *J. Insect Conservation* **17**, 1237–1253 (2013).
95. Narita, S. et al., *Wolbachia*-induced feminisation newly found in *Eurema hecabe*, a sibling species of *Eurema mandarina* (Lepidoptera: Pieridae). *Ecol. Ent*. **36**, 309–317 (2011).
96. Thipaksorn, A., W. Jamnongluk, P. Kittayapong, Molecular evidence of *Wolbachia* infection in natural populations of tropical Odonates. *Current Microbiology* **47**, 314–318 (2003).
97. Sekeyová, Z. et al., Identification of *Rickettsia africae* and *Wolbachia* sp. in *Ceratophyllus garei* fleas from passerine birds migrated from Africa. Vector-Borne and Zoonotic Diseases **12**, 539–543 (2012).

98. Heukelbach, J., I. Bonow, L. Witt, H. Feldmeier, P. Fischer, High infection rate of *Wolbachia* endobacteria in the sand flea *Tunga penetrans* from Brazil. *Acta Tropica* **92**, 225–230 (2004).
